# Identification of protein candidates in spermatozoa of water buffalo (*Bubalus bubalis*) bulls helps in predicting their fertility status

**DOI:** 10.3389/fcell.2023.1119220

**Published:** 2023-02-20

**Authors:** Seema Karanwal, Ankit Pal, Jatinder Singh Chera, Vipul Batra, Arumugam Kumaresan, Tirtha K. Datta, Rakesh Kumar

**Affiliations:** ^1^ Animal Genomics Laboratory, Animal Biotechnology Centre, National Dairy Research Institute, Karnal, India; ^2^ Theriogenelogy Laboratory, SRS of National Dairy Research Institute, Bengaluru, India

**Keywords:** spermatozoa, fertility, conception rate, proteomics, differentially abundant proteins

## Abstract

The water buffalo (*Bubalus bubalis*) is an indispensable part of the Indian dairy sector and in several instances, the farmers incur economic losses due to failed pregnancy after artificial insemination (AI). One of the key factors for the failure of conception is the use of semen from the bulls of low fertilizing potential and hence, it becomes important to predict the fertility status before performing AI. In this study, the global proteomic profile of high fertile (HF) and low fertile (LF) buffalo bull spermatozoa was established using a high-throughput LC-MS/MS technique. A total of 1,385 proteins (≥1 high-quality PSM/s, ≥1 unique peptides, *p* < 0.05, FDR < 0.01) were identified out of which, 1,002 were common between both the HF and LF groups while 288 and 95 proteins were unique to HF and LF groups respectively. We observed 211 and 342 proteins were significantly high (log Fc ≥ 2) and low abundant (log Fc ≤ 0.5) in HF spermatozoa (*p* < 0.05). Gene ontology analysis revealed that the fertility associated high abundant proteins in HF were involved in spermatogenesis, sperm motility, acrosome integrity, zona pellucida binding and other associated sperm functions. Besides this, the low abundant proteins in HF were involved in glycolysis, fatty acid degradation and inflammation. Furthermore, fertility related differentially abundant proteins (DAPs) on sperm *viz.*, AKAP3, Sp17, and DLD were validated through Western blotting and immunocytochemistry which was in coherence with the LC-MS/MS data. The DAPs identified in this study may be used as potential protein candidates for predicting fertility in buffaloes. Our findings provide an opportunity in mitigating the economic losses that farmers incur due to male infertility.

## 1 Introduction

Male fertility can be defined as the capability of spermatozoa to fertilize an oocyte and promote pregnancy which is crucial for the entire livestock (De Oliveira et al., 2013). Bull fertility plays a vital role in preserving herd genetics and productivity of livestock because a single semen ejaculate can be used to fertilise thousands of females via artificial insemination (Parisi et al., 2014). A single bull, on an average, produces a substantial number of sperms (approximately 4.8 billion sperms per ejaculate) in terms of normal morphology and motility, however, some bulls exhibit low sperm count with normal morphologies which render them sub-fertile (Guzick et al., 2001). Fertility disparities among individual bulls result in poor reproductive performance (Kastelic, 2013). Poor pregnancy rates have been observed due to artificial insemination of buffaloes with sperms from low fertile bulls. This raises the expense of maintaining such bulls and cows that do not exhibit pregnancy due to failed insemination (Parisi et al., 2014). Therefore, it is essential to investigate the male fertility in order to identify the factors leading to subfertility which could potentially reduce the agriculture economic losses. Several tests have been developed to predict the fertility status of bulls. Fluorescent dyes-based test has been developed over the past few decades but its applicability in assessing bull fertility is inconsistent and limited (Saacke et al., 2000; Kumaresan et al., 2017). The sperm functional test and sperm-oviduct binding assay are also used for buffalo bull fertility evaluation (Singh et al., 2016; Saraf et al., 2019). Spermatozoa contain many biomolecules such as lipids, glycans, nucleic acids, carbohydrates, lectins and proteins, and alterations in any of these biomolecules can potentially cause subfertility or infertility (Clark, 2013; Xin et al., 2014; Batra et al., 2020; Tomoiaga et al., 2020; Shan et al., 2021). Apart from these biomolecules, various factors like environment, nutrition, etc. may be responsible for male subfertility (Giahi et al., 2016; Fair and Lonergan, 2018). According to several studies, chromatin of sperm is modified during the developmental stage of spermatozoa in such a way that most of the histone proteins are supplanted by protamines (Miller et al., 1999; Grunewald et al., 2005; Miller and Ostermeier, 2006; Ren et al., 2017; Selvaraju et al., 2018). The exchange of histone to protamine in sperm chromatin ceases the gene transcription potential (Sillaste et al., 2017). It is also hypothesised that mature spermatozoa are not able to synthesize proteins (Rahman et al., 2013). Since sperm is a terminally differentiated cell without transcriptional and translational machinery and because spermatozoa carry pre-synthesized proteins as final products, therefore, proteomics is superior to other “omics” strategies for identifying the fertility biomarkers (Rahman et al., 2013; Sun et al., 2021; Hamilton et al., 2022). Several proteins are known to regulate male fertility by assisting the spermatozoa in maintaining their structure, survival, and fertilizing capability in the female reproductive tract (FRT). Very few studies have catalogued the proteomic repertoire of buffalo bull spermatozoa in past few years using 2D- DIGE, MALDI-TOF, and LC-MS/MS but they found only few proteins that were differentially abundant in contrasting fertility bulls (Muhammad Aslam et al., 2019; Binsila et al., 2021). Therefore, this study was designed i) to identify and quantitate the abundance of proteins in the buffalo spermatozoa obtained from the bulls of contrasting fertilizing abilities ii) to assess the functional significance of differentially abundant proteins (DAPs) in high and low fertile bull spermatozoa with *in situ* validation of some candidate proteins in distinct fertility groups of spermatozoa.

Herein, the raw mass spectra data searched against Uniprot *BOVINE reference* proteome database using Sequest search engine provided more than 1,000 proteins, out of which 553 proteins were found to be differentially abundant (≥1 high-quality PSM/s, ≥1 unique peptides, *p* < 0.05, FDR < 0.01) in high and low fertile buffalo bulls. The DAPs reported here such as AKAP3, AKAP4, Sp17, ACE3, PDIA3, DLD, etc. have roles in regulation of various sperm functions. These DAPs could be responsible for the differential fertility of buffalo bulls used in this study, nevertheless, further studies are warranted to elucidate their molecular functions in regulation of fertility.

## 2 Materials and methods

### 2.1 Chemicals and plasticware

All chemicals and reagents were procured from Sigma Aldrich Chemical Co., Ltd. (United States) unless stated otherwise. All plasticware was procured from Nunc Inc. (Thermo Scientific, United States).

### 2.2 Buffalo bull classification and semen straws collection

**FIGURE 1:**
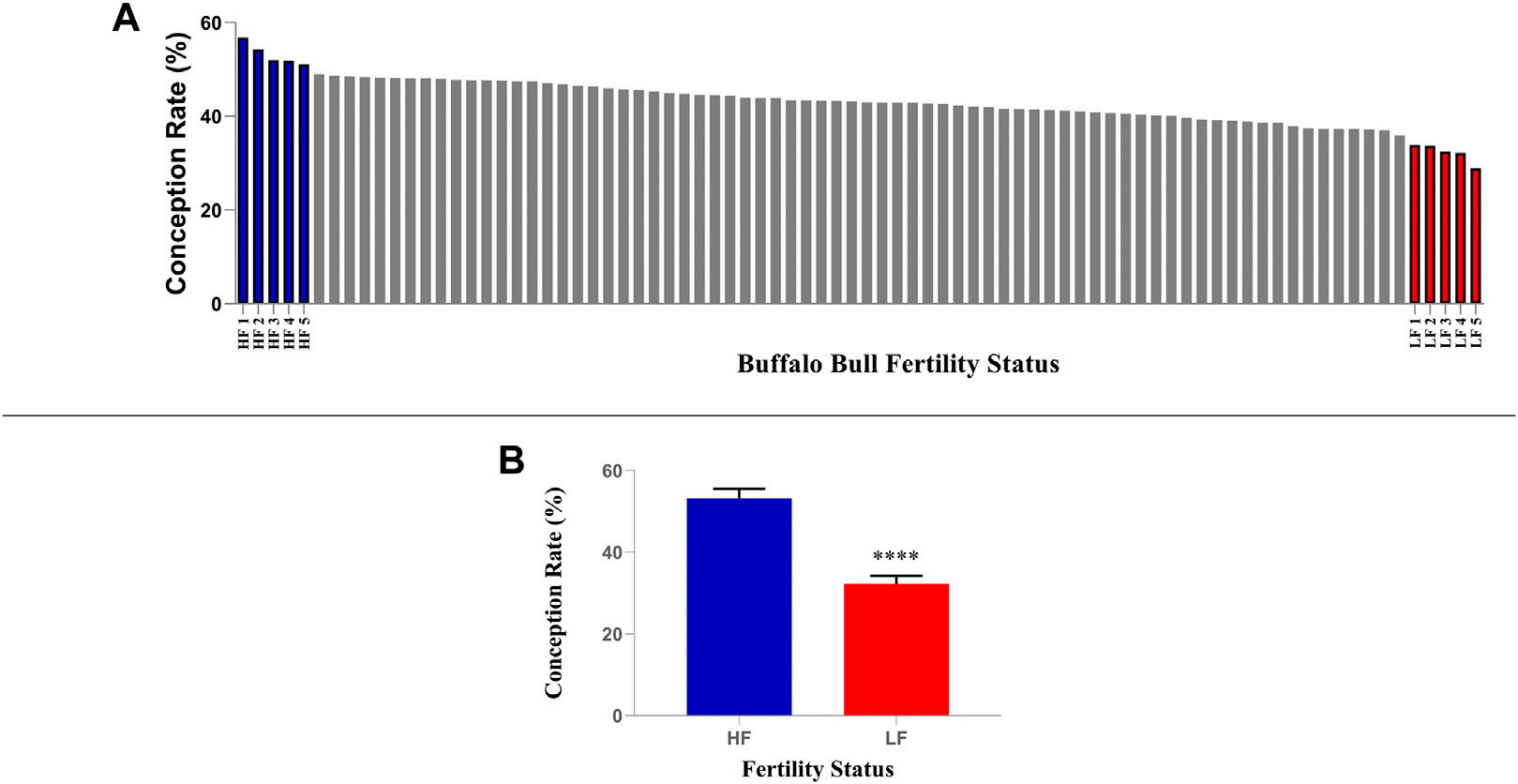
Selection of high and low fertile buffalo bulls based on conception rate (CR). **(A)** Five bulls with highest CRs were considered as high fertile while five bulls with lowest CRs were considered as low fertile. **(B)** The mean CRs of shortlisted high and low fertile bulls were significantly different (*p* < 0.0001).

More than eighty Murrah buffalo bulls having more than 50 insemination records were considered for the classification based on fertility (Figure 1). All of these bulls were maintained as per a regular feeding and management plan, and they were selected into the progeny testing programme following evaluation of their breeding soundness, semen quality factors such as semen volume, sperm count, viability and progressive motility. The conception rates (CRs) data was obtained from Artificial Breeding Research Center (ABRC), NDRI, India, and Central Institute for Research on Buffaloes (CIRB) Hisar, Haryana, India. The CRs of the 82 bulls considered in this study were subjected to Shapiro-Wilk normality test and they were found to fit into normal distribution (*p*-value = 0.781 where the null hypothesis of data fitting into a normal distribution was accepted) with a mean of 43.06% and a standard deviation (S.D) of 5.05%. Hence, the bulls with CRs lying between 48.11% and 38.01% were considered as average fertile. Ten buffalo bulls (*Bubalus bubalis*, *n* = 10), five each in high-fertility (HF, *n* = 5, CR between 51% and 56.7%) and low fertility (LF, *n* = 5, CR between 28.8% and 33.8%) chosen for the study had their CRs above and below the Mean ± 1 S.D (HF > 48.11% and LF < 38.01%) (Verma et al., 2014; Batra et al., 2020). The frozen semen (contained in 0.25 mL straws) of these bulls were used under a multi herd progeny testing program in India which were randomly assigned for breeding buffaloes in different herds maintained with uniform feeding and management schedule.

### 2.3 Plasma membrane integrity

Carboxy fluorescein diacetate (CFDA) (λ_ex_ 498 nm and λ_em_ at 517 nm) used in conjugation with propidium iodide (PI) (λ_ex_ 493 and λ_em_ 636 nm) (Sigma Aldrich, Germany), as previously described, was used to examine the sperm plasma membrane integrity and viability (Singh et al., 2016). The semen straws were thawed by submerging them in a water bath set at 38°C for 30 s, and the sample was then placed in a 15 mL centrifuge tube with 2 mL of working (1X) Sperm Tyrode’s albumin lactate pyruvate (Sp-TALP) (50 mM NaCl, 5 mM HEPES, 1.55 mM KCl, 0.2 mM EDTA, 0.2 mM MgCl_2_.6H_2_O, 0.15 mM NaH_2_PO_4_.2H_2_O, 1 mM 60% Na-lactate, and 0.98 Mm Na-Pyruvate). In a nutshell, filtered Sp-TALP was used to perform two sperm-washes on a frozen semen sample. First, 10 million spermatozoa were mixed with 50 µL of CFDA (0.5 mg/mL), and the mixture was incubated at 37°C for 15 min in the dark. After incubation, 2 µL of PI (0.3 mg/mL) was added, and the mixture was again incubated for 2 min. A thin smear was created on a microscopic slide using the pellet obtained after centrifugation at 800 g for 3 min. Antifading agent 1,4-diazabicyclo [2.2.2] octane (DABCO) was added on the smear and subsequently, a cover slip was placed carefully to avoid bubbles. Spermatozoa were examined using fluorescence microscope (Olympus IX73) with FITC (λ_ex_ 510–560 nm and λ_em_ 505 nm) and TRITC (λ_ex_ 550 nm and λ_em_ at 580 nm) filters at a ×60 magnification (Fig)A final image was produced by combining the images from the two filters.

### 2.4 Acrosomal integrity test

In order to determine the acrosome status of spermatozoa, fluorescein isothiocyanate conjugated peanut agglutinin (FITC-PNA) (λ_ex_ 494 nm and λ_em_ at 517 nm) was added in conjugation with propidium iodide (PI) (Singh et al., 2016). In a nutshell, 10 million spermatozoa were treated with 5 µL of FITC-PNA (25 μg/mL), and were then incubated at 37°C for 15 min in the dark. After incubation, 1 µL of PI was added and incubation was carried out for 2 min. Approximately, 200 µL 1X Sp-TALP was added to the samples and then centrifuged at 800 g for 3 min. After removing the supernatant, a thin smear was created on a slide from the pellet obtained. To minimize fluorescence quenching, a fading inhibitor (DABCO) was applied on top of the dried smear. Spermatozoa were examined by fluorescent microscopy (Olympus IX73) at ×60 magnification with and TRITC filters. A final image was created by combining images from the two filters.

### 2.5 Pre-processing of semen and protein extraction

The semen straws were thawed by submerging them in a water bath set at 38°C for 30 s, and the pooled samples of both the groups were then placed in a 15 mL centrifuge tube with 2 mL of working 1X Sp-TALP separately. The semen was washed 3X by centrifuging at 800 g for 10 min in Sp-TALP. After the last wash, the pellet was resuspended in 500 µL Sp-TALP and layered on top of 1 mL freshly prepared 50% percoll in 1X PBS (pH 7.2) to separate the spermatozoa from the seminal plasma that contains the non-sperm cells (such as bacteria or leucocytes). The sample was then centrifuged at 1,250 g for 20 min at 37°C using swinging bucket rotor. This procedure gathers spermatozoa that are motile and morphologically normal. To eliminate Percoll traces, motile spermatozoa were resuspended in 1 mL of 1X PBS and centrifuged for 5 min at 800 g at 4°C. The pellet obtained was re-suspended in RIPA buffer (approximately 100 µL/100 × 10^6^ spermatozoa) containing halt protease inhibitor cocktail (Selvam et al., 2019). Subsequently, the samples were kept at 4°C overnight to ensure complete lysis of the spermatozoa. Next day, supernatant (protein) was collected after centrifugation at 13,000 g for 30 min at 4°C and stored in −20°C. Isolated protein was quantified using the bicinchoninic acid protein test (Pierce™ BCA Protein Assay Kit cat no. 23225). Approximately, 120 µg protein from each individually pooled sample and ∼20 µg protein from each group was mixed with the 2X sample loading buffer (S3401) (5 µL β-mercaptoethanol + 95 µL SLB) and then denatured at 95°C for 5 min. Boiled samples were then loaded onto a pre-cast SDS-polyacrylamide gel and was run at 150 V under reducing conditions. Gel bands were visualized with Coomassie brilliant blue (R-250). To check the quality of the isolated protein samples, they were subjected to SDS-PAGE to check the integrity and possible degradation before the in-solution digestion. The proteins were found to be intact for both the pooled samples (Supplementary Figure S1).

### 2.6 In-solution digestion and peptides fractionation

The ∼100 µg protein left after the quality check of each pooled protein sample were purified using HiPPRTM Detergent Removal Spin Column Kit (Thermo Scientific TM). Lyophilized protein samples were alkylated with 100 mM iodoacetamide for 20 min, reduced with 45 mM dithiothreitol (DTT), and then was digested with trypsin (Promega, 1:50 trypsin/protein) overnight at 37°C. By adding 10% TFA, trypsin was inhibited. The high pH reversed-phase peptide fractionation kit (Pierce TM High pH Reversed-Phase Peptide Fractionation Kit, catalogue no. 84868) was used to separate 100 µg of protein into its constituent peptides based on their hydrophobicity thus, providing excellent orthogonality to low pH reversed-phase LC-MS gradients according to manufacturer instructions. The pooled peptides of each group were fractionated into eight fractions and each fraction was run with three technical replicates (8 fractions x 3 technical replicates = 24 total technical replicates for both HF and LF separately) in the mass spectrometry.

### 2.7 Mass spectrometry analysis of peptide mixture

The dried pellet was resuspended in buffer A (5% acetonitrile, 0.1% formic acid). The experiment was performed using EASY-nLC 1,000 system (Thermo Fisher Scientific) coupled to Thermo Fisher-*QExactive* equipped with nano-electrospray ion source. A total of 1.0 µg of the peptide mixture was resolved using 15 cm PicoFrit column (360 µm outer diameter, 75 µm inner diameter, 10 µm tip) filled with 2.0 µm of C18-resin (Dr Maeisch, Germany). The peptides were loaded with buffer A and eluted with a 0%–40% gradient of buffer B (95% acetonitrile, 0.1% formic acid) at a flow rate of 300 nL/min for 100 min. MS data was acquired using a data-dependent top10 method dynamically choosing the most abundant precursor ions from the survey scan. The mass spectrometry proteomics data have been submitted to the ProteomeXchange Consortium via the PRIDE (Perez-Riverol et al., 2022) partner repository with the dataset identifier PXD039680.

### 2.8 Protein identification and data analysis

All 48 samples (24 HF fractions and 24 LF fractions obtained) were processed and RAW files generated were analysed with Proteome Discoverer (v2.0) against the Uniprot Bovine reference proteome database. For Sequest search, the precursor and fragment mass tolerances were set at 10 ppm and 0.5 Da, respectively. The protease used to generate peptides i.e., enzyme specificity was set for trypsin/P (cleavage at the C terminus of “K/R: unless followed by “P”) along with maximum missed cleavages value of two. Carbamidomethyl on cysteine as fixed modification and oxidation of methionine and N-terminal acetylation were considered as variable modifications for database search. Both peptide spectrum match and protein false discovery rate were set  < 0.01 FDR (false discovery rate) to increase the confidence and remove the false-positive detection. The proteins abundances were further subjected to statistical analysis. Abundance matrices were filtered on the basis of valid quantification values. The filtered protein abundances were log2 transformed followed by missing values imputation using normal distribution. Student’s *t*-test was applied as the number of conditions were equal to two. The significance was calculated using *p*-values. All the proteins with *p*-values less than 0.05 were filtered and marked as significant proteins.

The statistically significant proteins were used for data visualisation. The log2 abundances were z-score transformed and visualised using Heatmap. The correlation coefficients, variance dimensionality analysis (PCA) were also calculated using these values. Proteins showing one or more than one unique peptide were considered for identification. The corresponding search against the database was obtained for all proteins from each group with a list of proteins along with its peak area-based quantification values, peptide spectrum match, score, coverage, number of unique peptides, number of peptides, number of peptide sequence matches, molecular weight, and the calculated pI.

### 2.9 Bioinformatics analysis of identified proteins

To understand the functional role of the proteins in spermatozoa, DAPs and unique proteins of both fertility groups were subjected to GO analysis and KEGG (Kyoto Encyclopedia of Gene and Genomes) pathway analysis. To identify the functions of unique proteins in HF and LF, the proteins present in at least two technical replicates were selected. The functional profiling of DAPs and unique proteins in sperms was carried out using Gene Ontology (GO) of the sperm proteome. GO was carried out using Uniprot and the Database for Annotation, Visualization, and Integrated Discovery (DAVID) gene enrichment tool v6.8. The proteins were then categrorized based on GO terms like molecular function (MF), biological function (BP), cellular component (CC) along with KEGG pathway enrichment. Top 10 BPs, MFs, and CCs and KEGG pathways were plotted in pie chart.

### 2.10 Western blotting for the shortlisted proteins

Sperm proteins isolated from high and low fertile semen straw were subjected to western blotting. The protocol followed has been described earlier (Selvam et al., 2019) with slight changes. In brief, 15 µg of protein from each group was electrophoresed in 12% SDS denaturing gel. The gel was equilibrated for 20 min in a transfer buffer before semi-dry transfer and the proteins were transferred to PVDF membrane (0.2 microns) 25 V, 2.5 A at room temperature for 20 min using power blotter station (Invitrogen). After transfer, the membrane was washed with Tris buffer saline containing Tween-20 (TBS-T) for 2 min and then incubated in a blocking buffer containing 5% bovine serum albumin (BSA) overnight at 4°C. The membrane was washed with TBS-T and incubated with primary antibodies namely, mouse anti-β-actin (Thermo Fisher Scientific, catalogue no. AM4302, 1:20,000 dilution), rabbit anti-Sp17 (Invitrogen, catalogue no. PA5-69981, 1:15,000 dilution), goat anti-AKAP3 (biorbyt, catalogue no. orb 18,844, 1:25,000 dilution) and rabbit anti-DLD (Invitrogen, catalogue no. PA5-27367, 1:15,000 dilution) at room temperature with constant rocking for 3 h. After incubation, the membrane was washed with TBS-T thrice for 10 min. Then, the membrane was incubated in HRP conjugated secondary antibodies such as goat anti-mouse for β-actin (Sigma, 1:70,000 dilution, catalogue no. 665739), goat anti-rabbit for Sp17 (Sigma, catalogue no. 632131, 1:90,000 dilution), rabbit anti-goat for AKAP3 (Abcam, catalogue no. ab6741, 1:150,000 dilution) and goat anti-rabbit for DLD (Sigma, catalogue no. 632131, 1:50,000 dilution) at room temperature for 1 h, followed by three washes (5 min each) in TBS-T. The membrane was then washed with TBS-T and then the immunocomplexes were detected using the ECL-HRP substrate (Sigma, WBULS0100). The membrane was incubated with substrate for 5 min in the dark and the signal was captured by the system-ray film (Fuji). The bands were analysed using ImageJ software (https://imagej.nih.gov/ij/). The β-actin (42 kDa) was used as normalizing control to obtain the relative abundance of Sp17 (22 kDa), AKAP3 (95 kDa), and DLD (54 kDa) proteins in sperm. From the list of DAPs, AKAP3, Sp17, and DLD were shortlisted based on high fold change in mass spectrometry data and presence of the protein in all replicates along with peptide matches. More importantly, these proteins were selected based on their involvement in the major pathways (Gene Ontology) and vital roles in regulating the sperm fertilizing capacity. Availability of the antibodies was also a criteria for selecting the three DAPs.

### 2.11 Localization of proteins on the spermatozoa using immunocytochemistry

Spermatozoa were isolated and washed with 1X PBS by centrifugation at 700 g at 4°C for 10 min. The sperm pellet was resuspended in 20 μL PBS and smear was prepared on a poly-L-lysine coated slide (Sigma-Aldrich). Cells were fixed using 4% paraformaldehyde in 1X PBS at 4°C for 20 min. Excess paraformaldehyde was rinsed off with PBS and then the sperms were permeabilised with permeabilization buffer (0.1% Triton X-100 in PBS) for 15 min at room temperature because the target protein was intracellular. The excess amount of permeabilization buffer was washed with PBS. The cells were incubated with 2% BSA for 1 h at room temperature to block the unspecific binding of the antibodies. Then slides were overlaid with a solution containing the primary antibody (same as those used in Western Blotting) AKAP3 (1:1,000), SP17 (1:250), and DLD (1:500) in blocking buffer (2% blocking buffer in 1X PBS) and incubated overnight at 4°C. Next day, solution was decanted and the cells were washed three times each for 5 min in 1X PBST (1X PBS with 0 .1% Tween 20). FITC labelled secondary antibody rabbit anti-goat for AKAP3 (1:1,000) (Invitrogen, catalogue no. 31533), goat anti-rabbit for SPA17 (1:500) and DLD (1:500) (Abcam, catalogue no. ab 97,050) were added in 2% BSA for 1 h at room temperature in the dark. Then, the slides were washed twice in PBST and counter staining was performed with 0.1 μg/mL DAPI (DNA stain, catalogue no. D9642) for 1 min and rinsed with 1X PBST. Coverslip was mounted with a drop of DABCO to visualize the cells under IX73 Olympus fluorescence microscope.

### 2.12 Statistical analysis

The sperm functional parameters and protein quantification through ImageJ were subjected to statistical analysis at 95% confidence level (*p* < 0.05) using the unpaired student’s *t*-test. The values have been expressed as mean ± SEM (Standard error mean).

## 3 Results

### 3.1 Sperm functional characteristics of bulls with contrasting fertility

**FIGURE 2:**
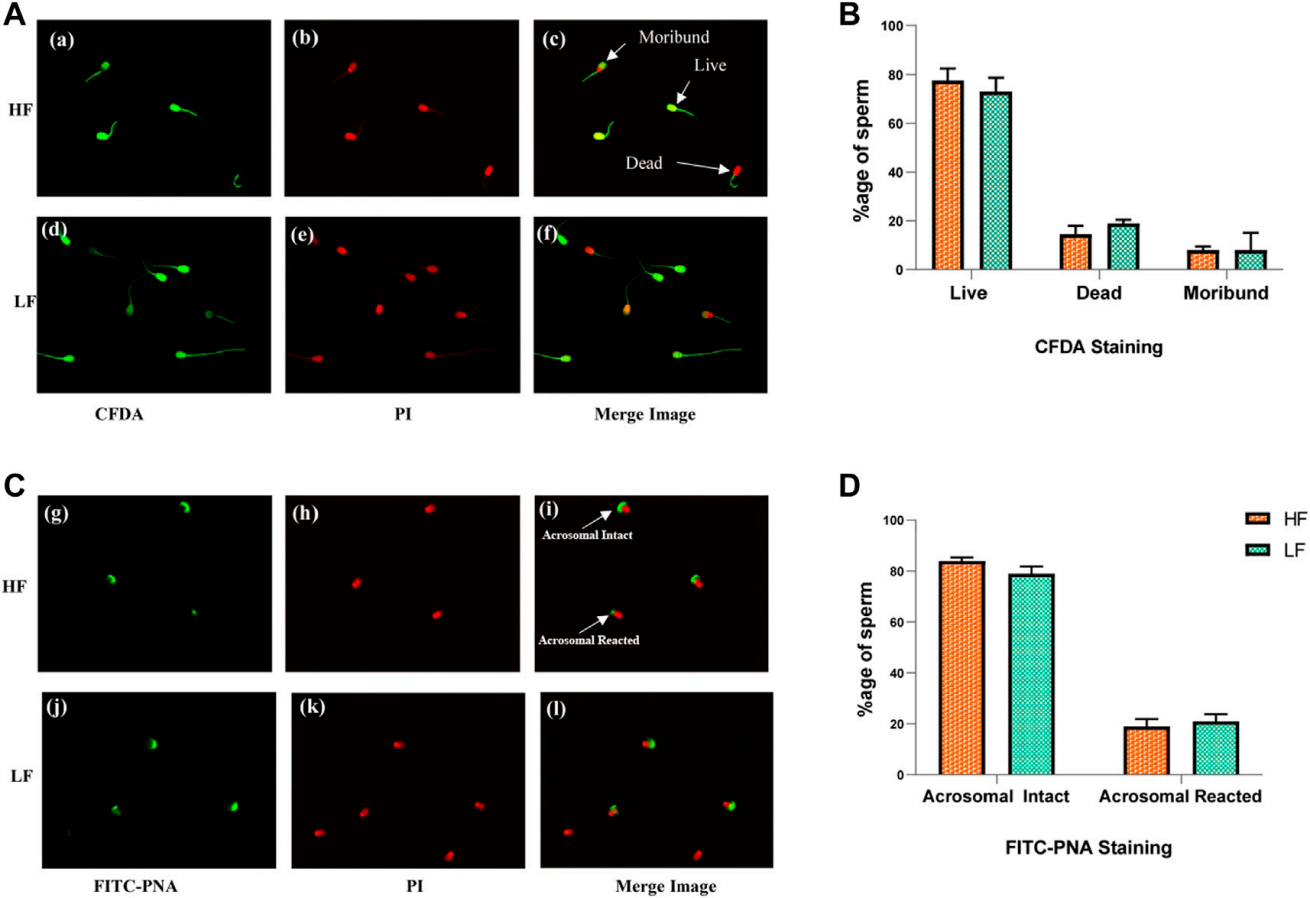
Sperm viability and acrosome integrity tests in HF and LF spermatozoa. **(A)** Cell viability assay through membrane integrity employing CFDA with PI was use to categorized sperms as live, dead and moribund in high fertile (a, b, c) and low fertile (d, e, f) groups. **(B)** Histogram plots showing percentage of different categories of sperms observed in the viability test. **(C)** Acrosome integrity test employing FITC- PNA along with PI was used to categorize the spermatozoa as acrosome intact or acrosome-reacted in high fertile (g, h, i) and low fertile (j, k, l) spermatozoa (original magnification 60X). **(D)** Histogram plots showing percentage of different categories of sperms observed in the acrosome integrity test.

The live-dead and acrosome integrity staining in spermatozoa were performed on individually pooled high fertile and low fertile semen samples (Figure 2). In live-dead staining, an average of 77.5% and 73% sperms were found live in high fertile (HF) and low fertile (LF) samples respectively. The average dead spermatozoa observed were 14.5% in HF and 19% in LF samples respectively. The moribund spermatozoa were 8% in both HF and LF respectively. Intense green fluorescence could be seen in sperms with their membranes intact, but bright red fluorescence was seen in spermatozoa that were dead. Moribund spermatozoa fluoresced in a combination of red and green giving an orange colour. After conducting the unpaired *t*-test, no significant difference (*p*-value > 0.05) was observed between HF and LF samples with respect to viability. In acrosome integrity test, we found 76% and 72% sperms with intact acrosomes in HF and LF samples respectively while 24% and 28% sperms were acrosome reacted in HF and LF respectively. Intense green colour along with red colour in sperm heads were considered as acrosome intact whereas sperm heads exhibiting red colour without intense green colour on top of the head were considered as acrosome reacted. After conducting the unpaired *t*-test, we observed no significant difference (*p*-value > 0.05) in acrosome integrity between HF and LF samples.

### 3.2 Proteomic profiling of high and low fertility bull spermatozoa

**FIGURE 3:**
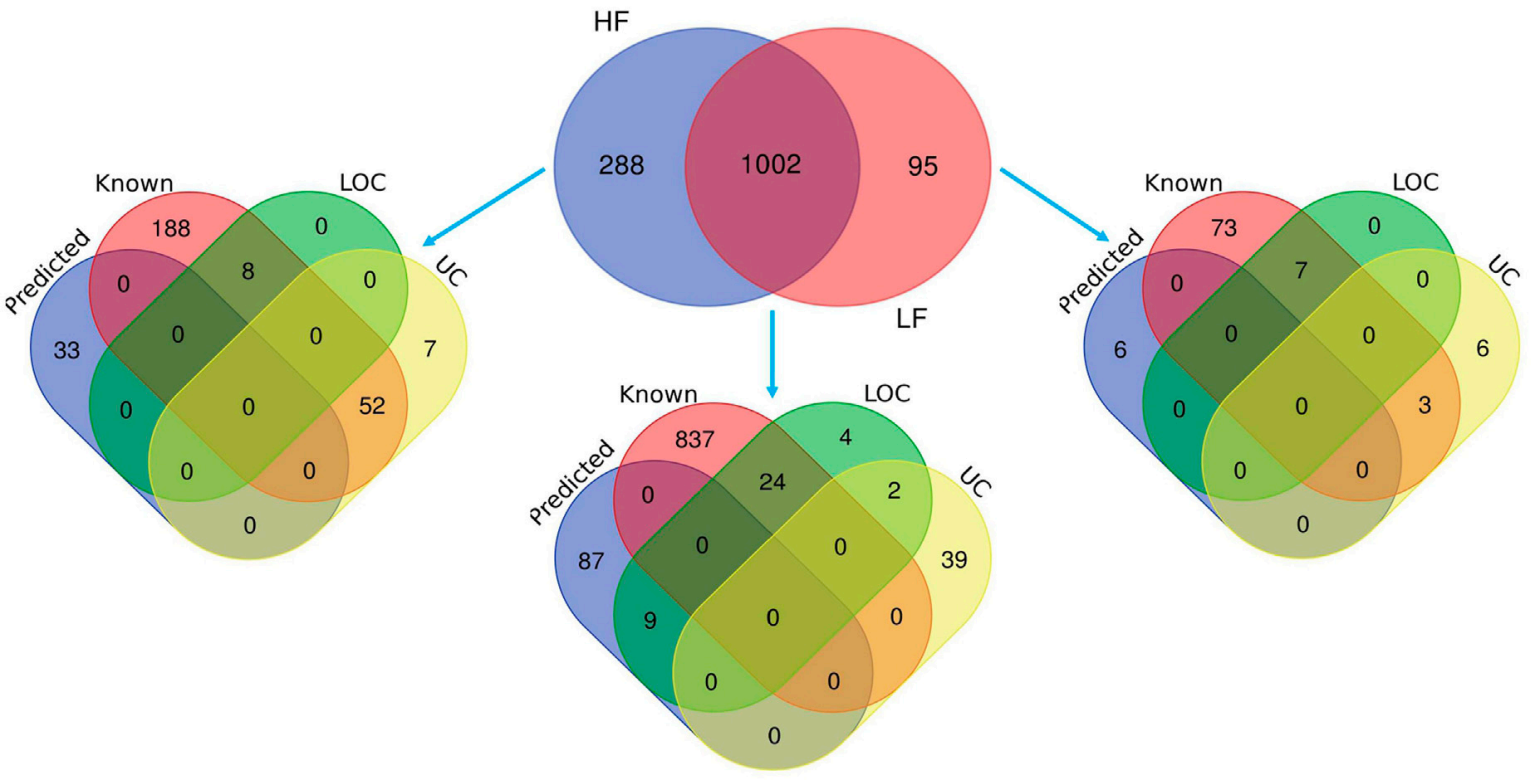
Venn diagrams representing the total identified proteins in HF and LF spermatozoa. The protein analytics tool Proteome Discoverer (v2.2) was used and 1,002 proteins were found common in both HF and LF sperms while 288 and 95 unique proteins were identified in HF and LF respectively with isoforms (*p* < 0.05, FDR < 0.01). Some of the proteins were known in nature, followed by predicted, uncharacterized (UC), and LOC proteins.

**FIGURE 4:**
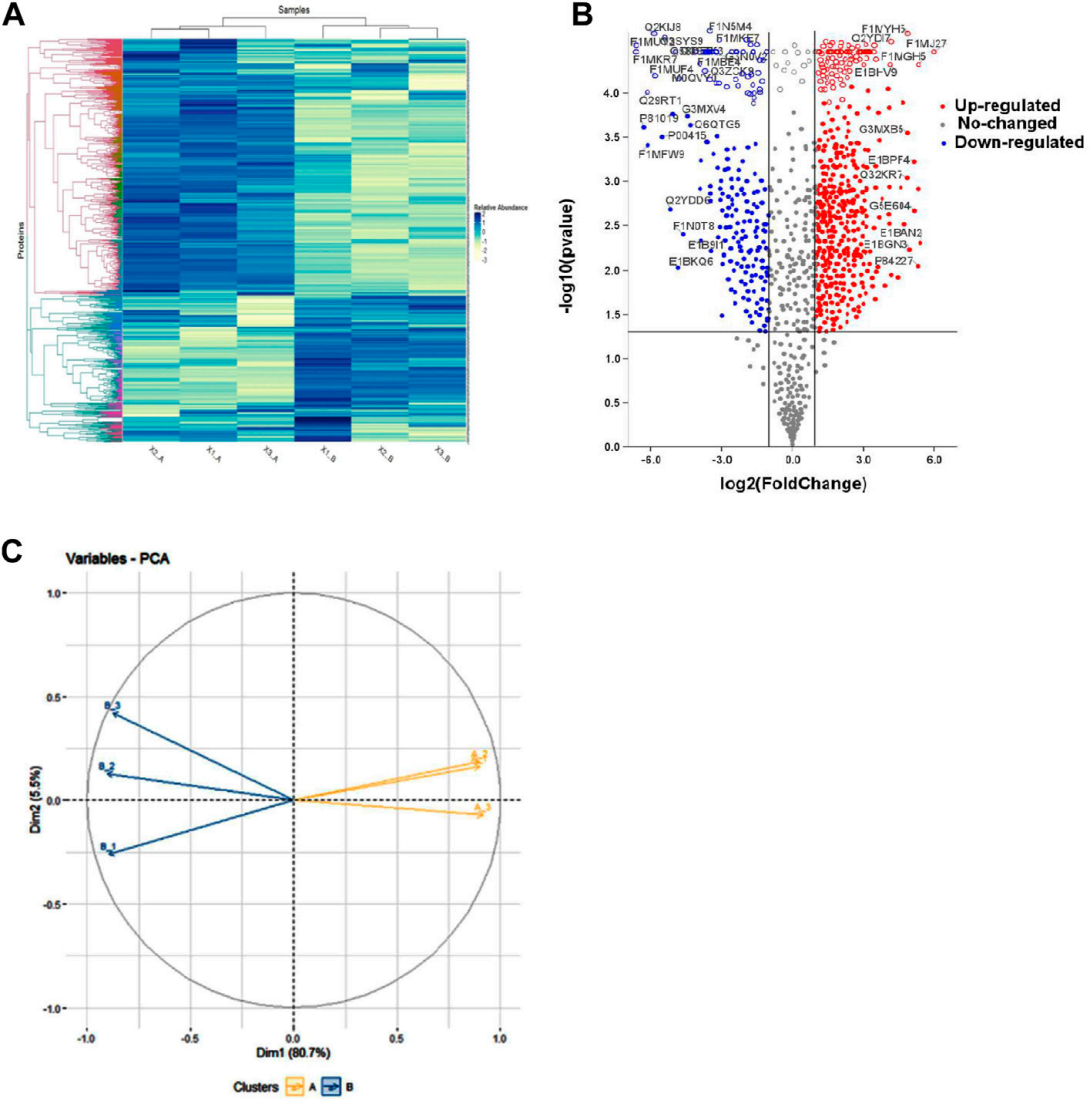
Representation of differentially abundant proteins (DAPs) in high and low fertile spermatozoa. **(A)** Heat map showing differentially abundant proteins (DAPs) among the replicates of HF and LF groups. Intense blue colour represents high abundance of proteins while white colour represents low abundance of proteins. **(B)** Volcano plot of all DAPs identified in the proteomics data determine by log fold change vs. –log10 *p*-value. Red points: DAPs that were significantly high abundant in high fertile bull (fold change >2; *p* < 0.05). Blue points: DAPs that were significantly low abundant in high fertile bull (fold change <0.5; *p* < 0.05). Grey points: DAPs that showed neutral abundance. Volcano plot showing the significantly abundant proteins determine by log fold change (log fold) vs. –log10 *p*-value. **(C)** PCA plot representing the level of variances between the replicates of HF and LF samples.

Mass spectrometry (MS) data analysis revealed that total, 1,385 proteins were identified in buffalo spermatozoa, out of which HF and LF groups had 1,290 and 1,097 proteins respectively (≥1 high-quality PSM/s, ≥1 peptides, *p* < 0.05, FDR < 0.01). Among these, 1,002 were common to both the groups, categorized as differentially abundant proteins (DAPs), while 288 and 95 proteins were unique to HF and LF groups respectively (Figure 3). We identified 553 proteins as significantly differentially abundant between the HF and LF spermatozoa (Figure 4). In spermatozoa, 211 proteins were high abundant (log fold change or Fc ≥ 2) while 342 proteins were low abundant (log Fc < 0.5) in HF w.r.t to LF spermatozoa (Tables 1, 2).

**TABLE 1:** List of fertility associated proteins highly abundant in high fertile bulls along with their functions and log fold changes.

S.No.	Gene	Description	*p*-value	Log 2 fold change	Function	References
1.	GSTM3	Glutathione S-transferase	0.002,119,403	2.16	It is an antioxidant enzyme.	Llavanera et al. (2020)
2.	PDIA3	PDIA3 protein	0.000397059	2.3	It is involved in human spermatozoa–zona pellucida (ZP) interaction.	Wong et al. (2017)
3.	ALDH2	Aldehyde dehydrogenase, mitochondrial	0.038430745	2.32	ALDH2 protect from oxidative stress, also help in progressive motility.	Gibb et al. (2016)
4.	SPA17	Sperm surface protein Sp17	0.00036	2.47	It is required spermatozoa–zona pellucida (ZP) during fertilization.	Grizzi et al. (2003)
5.	ATP2B1	Calcium-transporting ATPase	0.006257822	2.69	It is required for sperm motility.	Tempel and Shilling (2007)
6.	CRISP2	Cysteine rich secretory protein 2	0.026006791	2.8	CRISP2 are associated with the proposed role of this protein in gamete interaction.	Brukman et al. (2016)
7.	MNS1	Meiosis-specific nuclear structural protein 1	0.006784983	2.81	MNS1 is essential for spermiogenesis, the assembly of sperm flagella, and motile ciliary functions.	Zhao et al. (2013)
8.	IQCD	IQ domain-containing protein D	0.002003854	3.05	IQ motif containing D (IQCD), a new acrosomal protein involved in the acrosome reaction and fertilisation.	Zhang et al. (2022a)
9.	VAMP1	Vesicle-associated membrane protein 1	0.006361858	3.1	It is involved in membrane fusion during the acrosome reaction.	Rodriguez-Martinez et al. (2006)
10.	TSGA10	Testis specific 10	0.001257143	3.14	It is a key protein in spermatid differentiation/maturation process.	Asgari et al. (2021)
11.	SMRP1	Spermatid-specific manchette-related protein 1	0	3.16	Play a role in spermatid elongation required for correct head shape formation as well as sperm tail and acrosome formation.	Storey (2008)
12.	100851075	Outer dense fiber protein 2	0	3.3	ODFs are beneficial for sperm motility via stabilization of the axoneme and that hypo-expression of Odf family proteins is involved in the pathogenesis of asthenozoospermia.	Zhao et al. (2013)
13.	HSPA6	Heat shock protein family A (Hsp70) member 6	0.006281407	3.31	It stimulates sperm motility in Japanese quail (*Coturnix japonica*).	Verma et al. (2014)
14.	GSN	Gelsolin	0.008833333	3.35	Sperm capacitation requires actin polymerization, whereas F-actin must disperse prior to the acrosome reaction. It is an actin-severing protein, gelsolin, is inactive during capacitation and is activated prior to the acrosome reaction.	Finkelstein et al. (2010)
15.	AKAP3	A-kinase anchor protein 3	0.000341772	3.68	It maintains the integrity of subcellular structure of spermatozoa and regulate motility.	Xu et al. (2020)
16.	AKAP4	A-kinase anchoring protein 4	0	3.78	It is the most abundant protein in sperm FS, necessary for sperm motility.	Zhang et al. (2022b)
17.	HEX	Beta-hexosaminidase	0.001302961	3.99	Participation of β-hexosaminidase in human sperm–zona pellucida interaction.	Mita et al. (2005)
18.	eEF1a1	Elongation factor 1-alpha	0	4.052	It is crucial for spermatogenesis and male fertility in the Nile tilapia.	Cheng et al. (2009)
19.	SPATA3	Spermatogenesis associated 3	0.00487924	4.06	SPATA3 expressed in acrosome deletion in this gene cause hypo fertility in mice.	Giahi et al. (2016)
20.	BCAP31	B-cell receptor-associated protein 31	0.001384988	4.116	It is involved in spermatogenesis.	Guan et al. (2010)
21.	SPATA19	Spermatogenesis-associated protein 19, mitochondrial	0.003	4.233	This protein plays an important role in sperm motility by regulating the organization and function of the mitochondria.	Muralimanoharan et al. (2015)
22.	TCTE3	t-complex-associated-testis-expressed 3	0.001680328	4.272	Regulate sperm motility and morphology.	Shan et al. (2021)
23.	NSF	Vesicle-fusing ATPase	0.011498542	4.28	NSF is present in membranes of human spermatozoa and localizes to the acrosomal region help in acrosome exocytosis.	Mitra et al. (2005)
24.	RGS22	Regulator of G-protein signaling 22	0.011555129	4.285	It is specifically expressed in the testis and is involved in spermatogenesis.	Wu et al. (2000)
25.	TPI1	Triosephosphate isomerase	0.014086796	4.353	Triosephosphate isomerase 1 (TPI1) is a member of the glycolytic pathway, which is a critical source of energy for motility in mouse sperm.	Dirami et al. (2013)
26.	TSN	Translin	0.006951631	4.381	Translin provides genomic stability and spermatogenesis.	Guan et al. (2009)
27.	CD59	CD59 molecule, complement regulatory protein	0	4.47	Immuno-protection in FRT.	Simpson and Holmes (1994)
28.	PRSS55	serine protease 55	0.000991643	4.496	PRSS55 is essential for the structural differentiation of sperm.	Zhu et al. (2021)
29.	SNAP23	Synaptosomal-associated protein	0.022051903	4.672	Required for exocytosis of acrosome in human sperm.	Tomoiaga et al. (2020)
30.	H2AFV	Histone H2A.V	0.000413793	4.741	It is essential for global DNA methylation reprogramming during early vertebrate development and that embryonic development.	Madakashira et al. (2017)
31.	H2AFZ	Histone H2A.Z	0.003416283	4.741	Spermatogenesis	Zhao et al. (2013)
32.	AK1	Adenylate kinase isoenzyme 1	0.000448133	4.745	Sperm motility.	Cao et al. (2006)
33.	GAPDHS	Glyceraldehyde-3-phosphate dehydrogenase, testis-specific	0.002985366	7.261	It is a sperm-specific glycolytic enzyme involved in energy production during spermatogenesis and sperm motility.	Megnagi et al. (2015)
34.	TEKT3	Tektin-3	0	7.51	It is required for progressive sperm motility in mice.	Roy et al. (2009)
35.	TEKT4	Tektin-4	0	7.956	Tektin 4 is required for progressive sperm motility in mice.	Roy et al. (2009)
36.	CSNK2A2	Casein kinase II subunit alpha’ (Fragment)	0.006262335	7.982	It expressed in late stages of spermatogenesis, and male mice in which Csnk2a2 has been disrupted are infertile, with oligospermia and globozoospermia (‘round-headed’ spermatozoa).	Xu et al. (2020)
37.	SPINK2	Serine peptidase inhibitor, Kazal type 2 (Acrosin-trypsin inhibitor)	0.000696697	8.088	SPINK2 is necessary to neutralize the action of acrosomal proteases shortly after their synthesis and before they can be safely stored in the acrosome where they normally remain dormant until their release during the acrosome reaction, in the absence of SPINK2, protease-induced stress initiates Golgi fragmentation contributing to the arrest of spermatid differentiation and their shedding from the seminiferous epithelium.	Kumaresan et al. (2017)
38.	IRGC	Interferon-inducible GTPase 5	0	8.977	IRGC1, a testis-enriched immunity related GTPase, is important for fibrous sheath integrity and sperm motility in mice.	Kaneda et al. (2022)
39.	Sep-12	Septin-12	0	10.988	SEPT12 orchestrates the formation of mammalian sperm annulus by organizing core octameric complexes with other SEPT proteins.	Kuo et al. (2015)
40.	OAZ3	Uncharacterized protein (Fragment)	0	14.678	It is essential for rigid connection of sperm tails to heads in mouse.	Tokuhiro et al. (2009)
41.	TEKT5	Tektin-5	0	16.411	TEKT5 plays an important role in flagella formation during spermiogenesis as well as being implicated in sperm motility.	Cao et al. (2011)

**TABLE 2:** List of fertility associated proteins low abundant in high fertile bulls along with their functions and log fold changes.

S.No.	Gene	Description	*p*-value	Log 2 fold change	Function	References
1.		Seminal plasma protein BSP-30 kDa	0.00036	−4.51	BSP-30-kDa increase the binding of epididymal sperm to epithelium.	Gwathmey et al. (2006)
2.	CD47	Leukocyte surface antigen CD47	0	−3.24	CD47 protect sperm from macrophage phagocytosis within the female reproductive tract.	Muralimanoharan et al. (2015)
3.	AQP7	Aquaporin-7	0.00433	−3.14	Crucial for sperm maturation and osmoregulation.	Delgado-Bermúdez et al. (2019)
4.	SPAG5	Sperm-associated antigen 5	0.00072	−2.73	Associate with the sperm tail axoneme.	Fitzgerald et al. (2006)
5.	ROMO1	Reactive oxygen species modulator 1	0	−2.38	ROMO1 is one of the most important proteins in the production of reactive oxygen species.	Amini et al. (2022)
6.	CAMK4	Uncharacterized protein (CAMK4)	0	−2.28	Spermatogenesis.	Wu et al. (2000)
7.	PROM1	Uncharacterized protein (PROM1)	0	−2.24	Spermatogenesis.	Fargeas et al. (2004)
8.	RNASE4	Ribonuclease, RNase A family, 4	0.00736	−2.05	Sperm maturation.	Cheng et al. (2009)
9.	FUNDC2	FUN14 domain-containing protein 2	0	−2.02	Sperm motility	Binsila et al. (2021)
10.	CCIN	Calicin	0.01318	−1.75	It maintance the shape of spermatozoa.	Zhang et al. (2022b)
11.	DLD	Dihydrolipoyl dehydrogenase	0.00135	−1.62	DLD is required for hamster acrosome reaction	Kumar et al. (2008)
12.	PLCZ1	1-phosphatidylinositol 4,5-bisphosphate phosphodiesterase zeta-1	0.00131	−1.46	PLCZ1 is the predominant sperm oocyte-activation factor.	Jones et al. (2022)
13.	SLC26A8	Testis anion transporter 1	0.00123	−1.17	Sperm motility.	Dirami et al. (2013)
14.	ACOT2	Uncharacterized protein (ACOT2)	0.00128	−1.14	It contributes to produce ATP.	Tang et al. (2014)
15.	ACRBP	Acrosin-binding protein	0	−1.1	ACRBP present upon the surface of the sperm head facilitates capacitation and the AR in the porcine.	Kato et al. (2021)
16.	CLGN	Calmegin	0.00833	0.17	It mediate the interactions between sperm and egg.	Ikawa et al. (1997)
17.	CFL1	Cofilin-1	0.04925	0.31	Sperm capacitation and acrosome reaction.	Megnagi et al. (2015)
18.	CD46	Membrane cofactor protein CD46	0.00037	0.44	It provides acrosomal stability in rodents spermatozoa.	Clift et al. (2009)
19.	SPACA1	Sperm acrosome membrane-associated protein 1	0.00351	0.5	SPACA1 facilitate sperm-egg fusion.	Fujihara et al. (2012)

### 3.3 Gene Ontology and pathway enrichment analysis

#### 3.3.1 Gene Ontology and pathway analysis of proteins high abundant in HF spermatozoa

**FIGURE 5:**
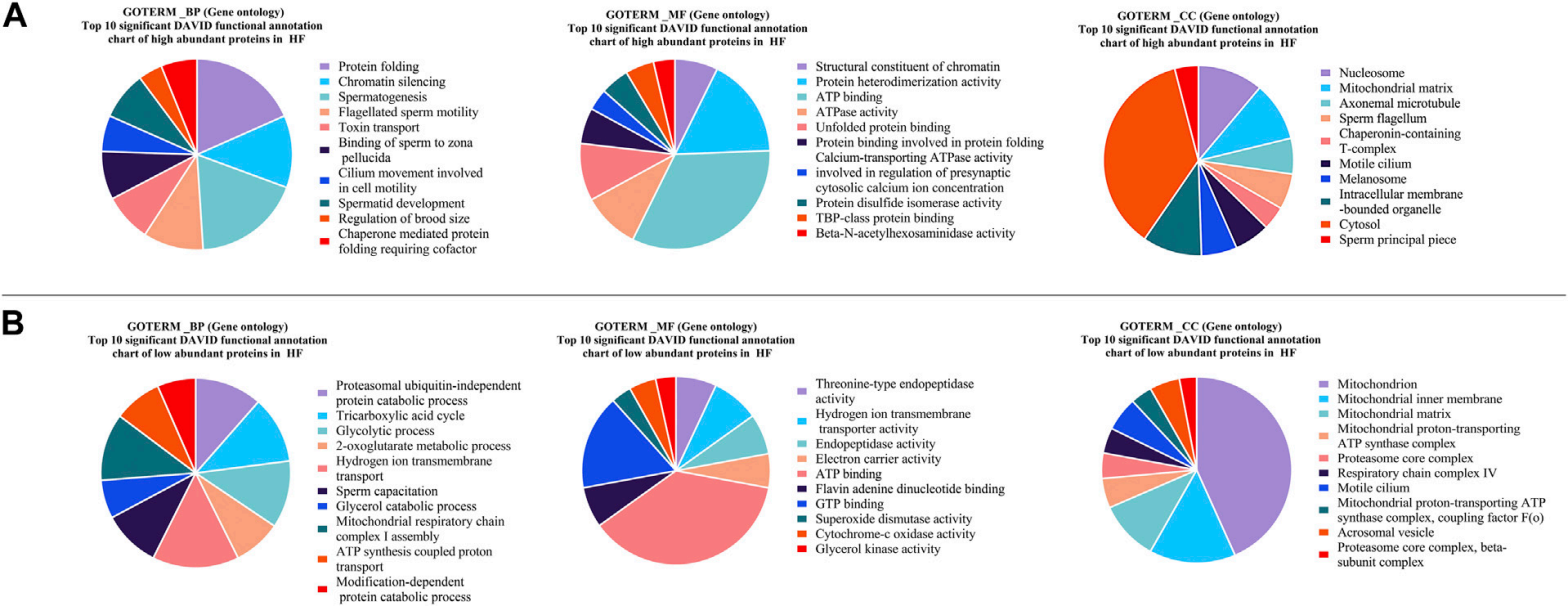
Pie charts representing top 10 Gene Ontology (GO) terms with respect to Biological Process (BP), Molecular Functions (MF) and Cellular Component (CC) **(A)** high abundant protein in High fertile **(B)** low abundant protein in High fertile spermatozoa.

GO analysis of the high abundant proteins (*p* < 0.05, log fold change ≥2) (Figure 5) revealed that the proteins associated with protein folding (CCT3, PDIA3, etc.; *p*-value = 1.84E-06), chromatin silencing (H2AZ1, H2AZ2, etc.; *p*-value = 1.75E-05), spermatogenesis (TSGA10, ODF2, SPATA19, OAZ3, SEPTIN12, etc.; *p*-value = 2.35E-04), flagellated sperm motility (AKAP4, etc.; *p*-value = 0.001), binding of sperm to zona pellucida (SPA17, etc.; *p*-value = 0.003), etc. were observed to be the enriched in terms of biological processes. The molecular functions of DAPs with highest level of significance were structural constituent of chromatin (H2AZ2, etc.; *p*-value = 7.85E-08), protein heterodimerization activity (P4HB, etc.; *p*-value = 3.11E-07), ATP binding (ATP2B2, etc.; *p*-value = 1.03E-06) and ATPase activity (KATNAL2, etc.; *p*-value = 1.29E-06). The proteins were enriched to be localized in nucleosome (LOC100297725, etc.; *p*-value = 2.43E-07), mitochondrial matrix (LYRM7, CLPP, etc.; *p*-value = 2.16E-06), axonemal microtubule (TEKT5, ODF2, etc.; *p*-value = 2.75E-06) and sperm principal piece (SPA17, AKAP4, AKAP3; *p*-value = 4.30E-04). The KEGG pathways associated with Neutrophil extracellular trap formation (LOC100297725, H3-5, etc.; *p*-value = 2.39E-05), and Metabolic pathways (TKFC, HEXA, etc.; *p*-value = 6.83E-4) etc. The fertility associated HF high abundant proteins along with their functions and log fold changes have been listed in Table 1.

#### 3.3.2 Gene Ontology and pathways analysis of proteins low abundant in HF spermatozoa

GO analysis of the low abundant proteins (*p* < 0.05, log fold change <0.5) (Figure 5) revealed that the proteins were associated with biological processes like proteasomal ubiquitin-independent protein catabolic process (PSMB5, etc.; *p*-value = 2.92E-07), tricarboxylic acid cycle (DLAT, SUCLG2, etc.; *p*-value = 2.90E-06) glycolytic process (PGAM2, etc.; *p*-value = 3.55E-06), sperm capacitation (DLD, etc.; *p*-value = 1.11E-05) etc. The molecular functions of DAPs were threonine-type endopeptidase activity (PSMB7, etc.; *p*-value = 3.25E-07), hydrogen ion transmembrane transporter activity (SLC25A5, ATP5MG, etc.; *p*-value = 1.13E-06), endopeptidase activity (PSMB7, etc.; *p*-value = 2.24E-04) and ATP binding (MYO18A, CARS2, etc.; *p*-value = 3.88-E04) etc. The proteins associated with cellular component were localized in mitochondrion (ROMO1, etc.; *p*-value = 9.73E-25), mitochondrial inner membrane (SLC25A11, FABP3, etc.; *p*-value = 5.52E-09) and mitochondrial matrix (FABP3, etc.; *p*-value = 1.86E-07). The KEGG pathways associated with these DAPs were Oxidative phosphorylation (UQCR10, ATP5F1E, etc.; *p*-value = 7.63E-14) and Carbon metabolism (DLD, etc.; *p*-value = 1.63E-12) etc.

Furthermore, it is crucial to note that high abundant proteins were linked to a variety of diseases like Pathways of neurodegeneration-multiple diseases (CSNK2A2, HSPA5, etc.; *p*-value = 5.34E-08) and Parkinson’s disease (LOC101906363, etc.; *p*-value = 3.94E-07). Moreover, Parkinson’s disease (NDUFA9, TUBB4B, etc.; *p*-value = 4.94E-21), Prion disease (UQCR10, SDHA, etc.; *p*-value = 4.82E-17) and Pathways of neurodegeneration - multiple diseases (UBA52, etc.; *p*-value = 6.44E-16) were linked to the low abundant proteins in HF (Supplementary Table S2; Figure 2).

#### 3.3.3 GO analysis of unique proteins in high fertile bull spermatozoa

GO analysis of the unique proteins from HF buffalo bull spermatozoa (Supplementary Figure S3) revealed that these proteins were associated with biological processes such as antigen processing and presentation of endogenous peptide antigen via MHC class Ib (BOLA, BOLA class I histocompatibility antigen, alpha chain BL3-7; *p*-value = 7.96E-12) and regulation of RNA splicing, FUS, HNRNPH2, etc.; *p*-value = 3.24E-09). Molecular function of these proteins were RNA binding (HNRNPH1, NONO, etc.; *p*-value = 6.27E-23) and structural constituent of ribosome (RPL37A, RPS24, etc.; *p*-value = 1.88E-11). In terms of cellular component, the proteins were found to be localized in the ribonucleoprotein complex (PCBP1, EEF2, etc.; *p*-value = 2.57E-14). KEGG Pathways of these protein were associated with Spliceosome (SRSF3, SF3B3, etc.; *p*-value = 2.48E-14), Ribosome (RPL18, RPS17, etc.; *p*-value = 8.40E-08), etc.

#### 3.3.4 GO analysis of unique proteins in low fertile bull spermatozoa

GO analysis of the unique proteins of LF buffalo bull spermatozoa (Supplementary Table S2) revealed that the proteins were associated with biological processes like cellular oxidant detoxification (LOC107131172, TXNRD2, etc.; *p*-value = 1.71E-09), negative regulation of chromatin silencing (H1-2, H1-3, etc.; *p*-value = 3.29E-07), etc. Molecular function of these unique proteins were organic acid binding (LOC107131172, HBE1, etc.; *p*-value = 1.45E-07), oxygen transporter activity (LOC107131172, etc.; *p*-value = 4.76E-07), etc. The cellular component analysis revealed that the proteins were found to be localized in haptoglobin-hemoglobin complex (LOC107131172, etc.; *p*-value = 1.40E-07) and hemoglobin complex (HBB, etc.; *p*-value = 1.95E-07). KEGG Pathways showed that the proteins were involved in prion disease (ND5, MT-ND3, etc.; *p*-value = 1.45E-06), Parkinson’s disease (MT-ND3, etc.; *p*-value = 1.45E-06), etc.

## 4 Validation of AKAP3, Sp17, and DLD through western blotting

**FIGURE 6:**
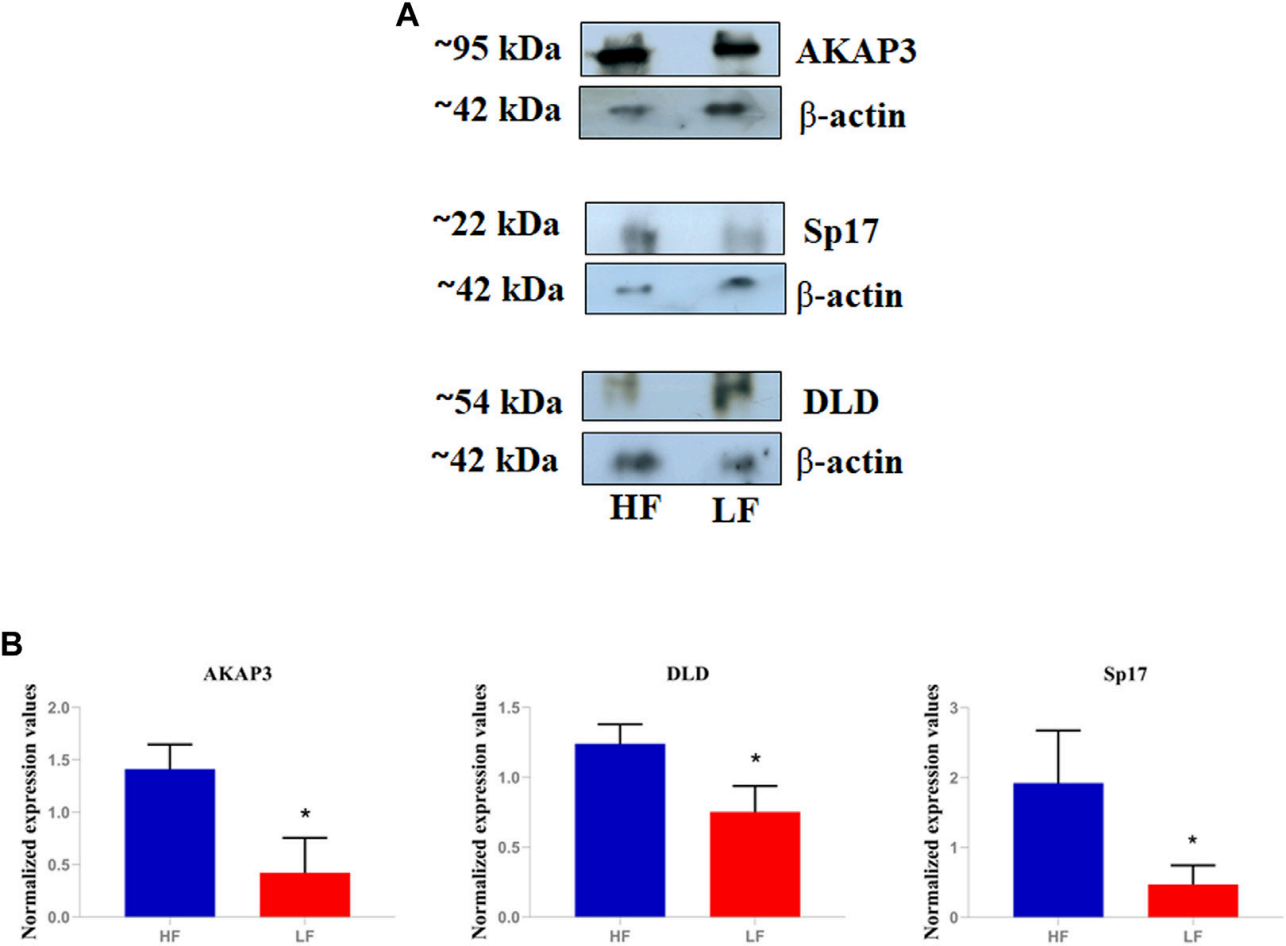
Validation of differential abundance of AKAP3, Sp17, and DLD using western blot. **(A)** The blot image showing relative abundance of AKAP3, Sp17, and DLD in HF and LF samples taking β-actin as calibrator. **(B)** Histograms representing the relative intensities of AKAP3, Sp17, and DLD among the HF and LF samples that were calculated using the blot images in the ImageJ software.

We performed the Western blotting of DAPs namely AKAP3, Sp17, and DLD (Figure 6). Western Blotting results revealed that the proteins namely AKAP3 (3.68- log fold) and Sp17 (2.47- log fold) were significantly (*p* < 0.05) high abundant in HF spermatozoa. Whereas abundance of DLD (−1.6 −log fold) was significantly (*p* < 0.05) low abundant in HF group when compared to LF group. The relative abundance of protein Sp17, DLD, and AKAP3 were normalized with ß-actin.

## 5 Cellular localization of AKAP3, SP17, and DLD through immunocytochemistry

**FIGURE 7:**
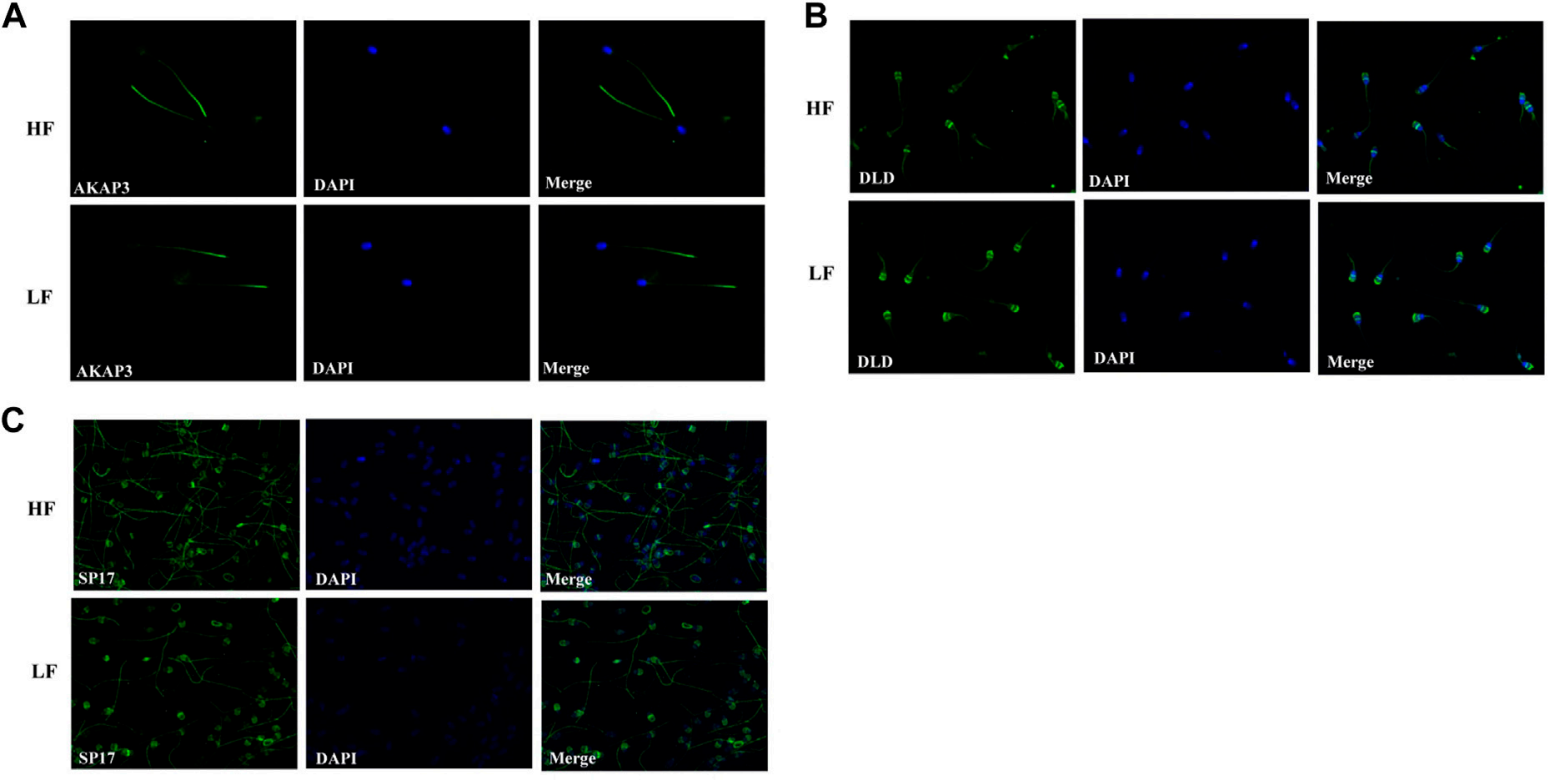
The cellular localization of **(A)** AKAP3, **(B)** DLD, and **(C)** Sp17 in HF and LF spermatozoa using FITC-labelled antibodies. Nuclear staining was carried out using DAPI. The images were captured at ×600 magnification.

AKAP3, SP17, and DLD were subjected to immunocytochemistry and it was observed that Sp17 was localised to the middle piece and in scattered patches all throughout the head area, whereas AKAP3 was located predominantly in the middle piece and scattered patches found in the lower tail of the spermatozoa of both, the HF and LF groups (Figure 7). Dihydrolipoamide dehydrogenase (DLD) was low abundant in the HF group and was localised in the acrosome along with the principal piece of flagella. Here, we only mapped the localization of these shortlisted proteins on the spermatozoa of distinct fertility bulls however, quantitation experiments were not performed.

## 6 Discussion

The present work was undertaken to find out whether spermatozoa from buffalo bulls with contrasting fertility differ in their protein abundance and profile. We hypothesized that the spermatozoa of high and low fertility bulls contain differential abundance of proteins that can affect the motility, immunity, capacitation, adhesion, acrosome reaction and zona pellucida binding which ultimately affect the fertilizing capacity. We aimed to decipher the differential abundance of proteins (DAPs) in HF and LF bulls’ spermatozoa and to investigate the effect of these proteins on fertility.

**FIGURE 8:**
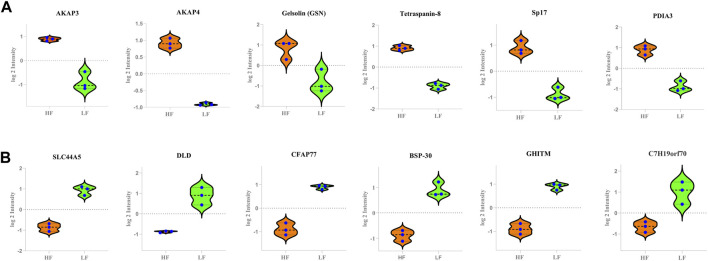
The Violin plots representing the relative abundance of various DAPs between the HF and LF groups. In the plots, y-axes represent log2 intensities of the respective DAPs. **(A)** high abundant protein in High fertile **(B)** low abundant protein in High fertile spermatozoa.

The buffalo bull sperm proteomic profile was decoded with the help of LC-MS/MS and computational biology. The whole sperm proteome (combined from both HF and LF samples) analysis revealed that the sperm contained 1,385 proteins. The existence of 288 and 95 unique proteins in high- and low fertile bull sperm, respectively highlights the fundamental variations in sperm functions that may compromise sperm fertility. Decoding the comparative proteomic profiling of HF and LF buffalo bull spermatozoa using high throughput LC-MS/MS pave ways to understand the associated pathways governing the sperm functionalties. Out of 553 DAPs, we shortlisted 20 DAPs that were significantly high and low abundant in HF that were intricately involved in the pathways regulating the vital sperm functional traits related to the fertilization process (Figure 8).

In terms of sperm functional tests such as viability and acrosome integrity, no significant differences were found among both the groups. Therefore, predicting bull fertility based on sperm functional characteristics may not be an efficient method (Barbăroșie et al., 2021) and can only be used as a means to determine semen quality (Rodríguez-Martínez, 2006). As a result, proteomic analysis of semen was conducted to evaluate the bull fertility status.

Before ejaculation, pre-mature spermatozoa go through a process called sperm maturation, which involves both changes in flagellar beating and the acquisition of traits needed to effectively confront the hostile female reproductive tract (FRT). Therefore, sperm development process should be error free. Biological process of high abundant proteins in HF were enriched with the Protein disulphide isomerase like PDIA1 and PDIA3 that regulate protein quality and redox regulation (Ellerman et al., 2006; Zhao et al., 2013; Wong et al., 2017; Chichiarelli et al., 2022) Sp17 regulates acrosomal reaction and interactions with the zona pellucida during the fertilization process (Chiriva-Internati et al., 2009). Motility is a unique property of spermatozoa because of which, it travels through the FRT to fertilise an egg. Therefore, both naturally occurring and aided conception depend greatly on motility. Some proteins associated with spermatogenesis process such as Testis specific 10 (TSGA10), Outer dense fiber protein 2 (ODF2), DNAJB13 are present in sperm tail and are responsible for cytoskeletal maintainenance and its formation along with regulation of motility (Guan et al., 2009; Guan et al., 2010; Li and Liu, 2014; Tajaddini Mahani et al., 2016; Lehti and Sironen, 2017; Asgari et al., 2021). Ornithine decarboxylase antizymes (OAZs) expressed specifically in germline cells, regulate polyamine concentration during spermiogenesis which is essential for cell proliferation and differentiation. OAZ-t mutant mice sperm showed loss of connection between the head and tail indicating that OAZ-t is essential for the formation of a rigid junction between the head and the tail during sperm development (Tokuhiro et al., 2009). Molecular functions of the high abundant proteins were enriched with the protein H3.5 histone, was expressed in human testis that regulates epigenetic changes important for normal spermatogenesis (Shiraishi et al., 2018). ATP binding proteins such as Katanin p60 ATPase-containing subunit A-like 2 (KATNAL2) regulates sperm maturation, head morphology and the structure of mitochondrial sheaths and flagella (Wei et al., 2021). Sperm capacitation and acrosomal reaction are the crucial functions that help the sperm to reach the site of fertilization and penetrate the zona pellucida in order to fuse with the oocyte membrane. HSPA5 influences the ability of sperm to engage in oocyte interactions in a calcium-dependent manner (Dun et al., 2012). In terms of cellular component, the proteins were mainly involved in motility (AKAP4, AKAP3, Testis specific 10, SPA17) which regulate the assembly of cytoskeleton in sperm tail (Gibb et al., 2016; Tajaddini Mahani et al., 2016; Zhang et al., 2022a).

The biological process of low abundant proteins in HF were enriched in glycolytic process which is in accordance with a previous report (Guo et al., 2019). The glycolytic pathway in humans is the main source of ATP production to sustain the energy needs for progressive motility and capacity (Hereng et al., 2011). Although, glycolysis is a pivotal process, however, excessive glycolytic activity might lower the intracellular pH which can impair capacitation in bull sperm (Storey, 2008). Immature and asthenozoospermic spermatozoa exhibit aberrant expression of sperm-specific glycolytic enzymes (PGK2) (Liu et al., 2019). Fatty acid binding protein3 (FABP3) is another protein found to be involved in inflammatory reaction. In our investigation, we discovered that FABP3 was low abundant in HF. Inflammatory reactions within the male genital tract are inevitably connected with oxidative stress (Azenabor et al., 2015; Wu et al., 2022) Oxidative stress, particularly on sperm, can lead to DNA damage and sperm death, which may lead to male infertility (Alahmar, 2019). Increased levels of reactive oxygen species (ROS) have been implicated as a cause of infertility (Wong et al., 2017). Overexpression of reactive oxygen species modulator 1 (ROMO1) protein in the mitochondria elevates the production of ROS (Cheng et al., 2009). However, optimum production of ROS is necessary for physiological activity and is essential for fertility.

Gene ontology analysis of unique proteins in HF revealed that they are associated with the immunity like BOLA class I histocompatibility antigen protein which is involved in providing resistance to several diseases such as mastitis and bovine leukemia virus (BLV)-induced lymphoma etc. BoLA is also known to influence milk yield, growth and reproduction (Takeshima and Aida, 2006). Many of the unique proteins in HF were also found to be involved in the splicing of pre-m-RNA and formation of spliceosome complex may be responsible for the protein translation required during capacitation in FRT (Will and Lührmann, 2011; Hoshika et al., 2019; Song et al., 2020). In this study, we found that the unique proteins in LF were enriched with disease related pathways such as prion disease, Parkinson’s disease, aging, etc. (Rottenberg and Hoek, 2021).

Quantitative proteomic analysis revealed that more than twenty proteins related to the fertility were significantly high and low abundant in HF spermatozoa. One of the significantly abundant proteins in high fertile group, AKAP3, was validated using western blotting and localized using immunocytochemistry in contrasting fertility groups. AKAP3 is highly essential for sperm motility and hypermotility to cross the pugnacious environment of FRT. Immunocytochemistry results clearly showed that AKAP3 was expressed all across the spermatozoa’s tail. AKAPs are pivotal proteins that commence the signalling cascade during capacitation process. These proteins are structurally diverse but conserved in signalling functions (Welling, 2012). Some AKAPs play crucial roles in gametogenesis both, in male and female reproductive systems as well as in other biological processes (Luconi et al., 2011). The perfect coordination of AKAP3 along with AKAP4 is required for the formation of sperm fibrous sheath which is essential for motility and hypermotility. Lack of AKAP3 causes global changes in the sperm proteome and mis-localization of sperm proteins, as well as displacement of PKA subunits in mature sperm which may be the underlying cause of immotility. Interestingly, sperms from AKAP3 null mice have been reported to lack fibrous sheath (Xu et al., 2020). In some circumstances, partial deletion of AKAP3 and AKAP4 that correspond to probable AKAP3/AKAP4 binding sites may be linked to dysplasia of the fibrous sheath (Baccetti et al., 2005). In humans, bicarbonate ions enhance sperm hyperactivation and motility by activating soluble adenyl cyclase and tyrosine phosphorylation of AKAP3 by PKA recruitment (Luconi et al., 2005). Therefore, in accordance with the previous reports, the current study suggests that AKAP3 might be involved in maintaining sperm motility.

Abundance of another protein in high fertile bulls, Sp17, was also validated through western blotting and its cellular localization through immunocytochemistry. Sp17 has been reported to be essential for zona pellucida binding during fertilization process (Chiriva-Internati et al., 2009). Immunocytochemistry results clearly showed that Sp17 was expressed all across the spermatozoa’s tail and in the post-acrosomal regions. Sp17, a low molecular weight sperm protein that is a member of the sperm autoantigens (RSA) family. It has been confirmed to interact with the zona pellucida carbohydrate component (Lea et al., 1998). The first RSA from a rabbit was identified, sequenced, and characterized as a 17-kDa mannose-binding protein (O’Rand et al., 1988). According to the previous reports, in many mammalian species, Sp17 was exclusively expressed in spermatozoa maturation phases i.e. spermatocytes, spermatids, spermatozoa, and seminiferous epithelium. No evidence of Sp17 expression was found in spermatogonia, Sertoli cells, Leydig cells or any other kind of somatic cell (Kong et al., 1995; Adoyo et al., 1997; Grizzi et al., 2003). Therefore, Sp17 may be involved in controlling sperm-zona pellucida binding in buffalo as well which is essential for fertilization.

**FIGURE 9:**
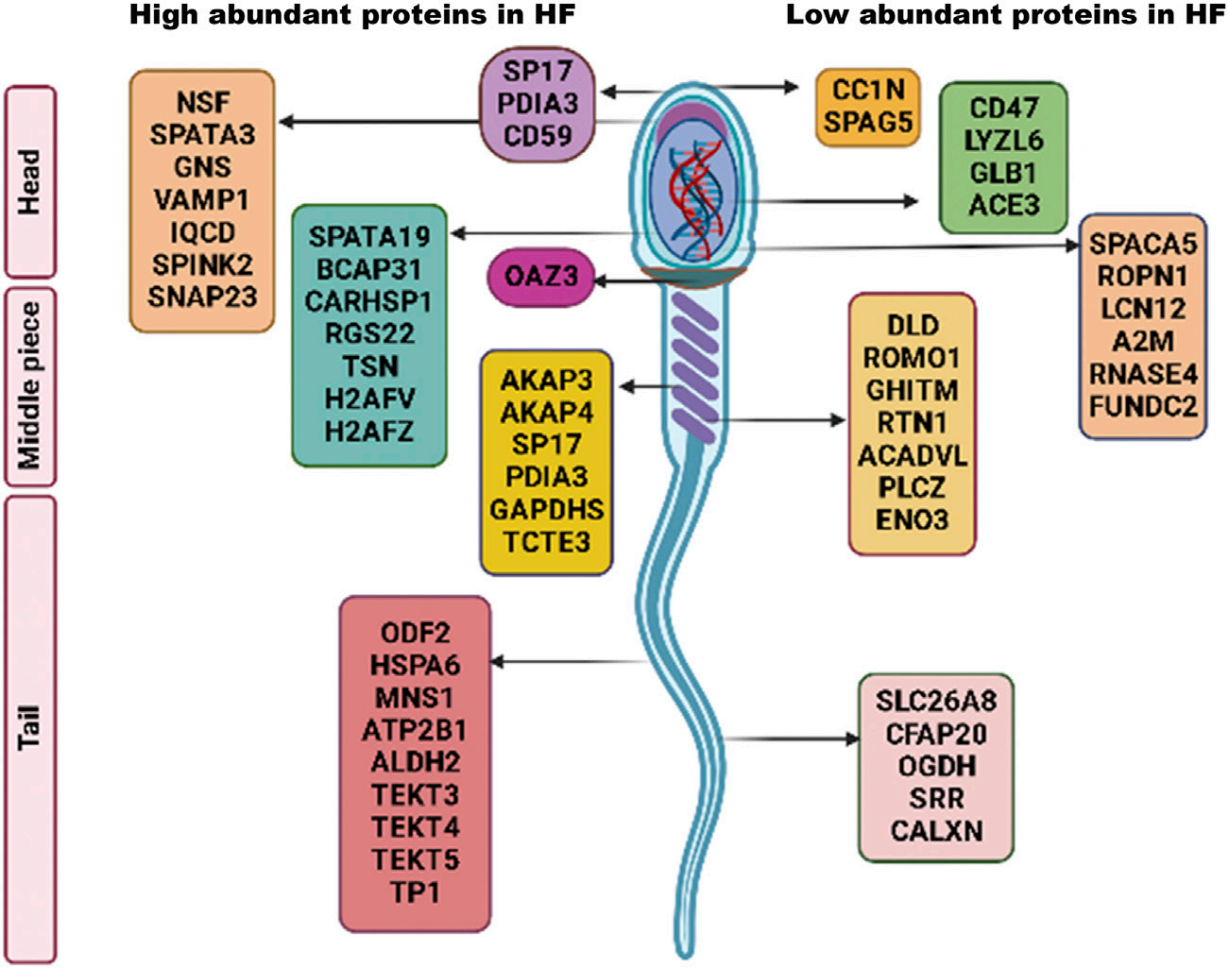
Schematic representation of fertility associated high and low abundant proteins in HF spermatozoa.

Dihydrolipoamide dehydrogenase (DLD) was found to be significantly low abundant in HF bull spermatozoa and immunocytochemistry results clearly showed that DLD was expressed in the middle piece of spermatozoa’s tail and in the acrosomal regions. DLD, an E3 subunit of the pyruvate dehydrogenase complex (PDH), found in the mitochondria is essential for the energy metabolism (Mitra and Shivaji, 2004). In this report, we found that this mitochondrial protein was found in spermatozoa tail and head region in addition to mid-piece which houses the mitochondria. Previous reports have also shown the non-canonical localization of DLD protein in Male Golden hamster spermatozoa (Mitra et al., 2005). DLD inhibition studies suggest that it is involved in the capacitation and acrosome reaction (Panneerdoss et al., 2012). In our results from sperm functional tests, we did not find significant difference in the number of acrosome reacted sperms between the HF and LF samples; so it is highly likely that DLD plays some other roles that may be involved in the deterioration of sperm quality. The fertility associated HF upregulated and downregulated proteins in spermatozoa with respect to localization have been represented in Figure 9. However, there are certain limitations in the study with respect to the number of samples considered and the sample preparation methods. The number of buffalo bulls under field fertility trials were very limited and it takes a long time to assess the field fertility of the bulls. Additionally, due to technical and financial constraints, the samples from individual bulls within the high and the low fertile group were pooled instead of considering individual samples for proteomics analysis and Western blotting. However, Pooling of the samples reduces the sample size while maintaining a high degree of confidence in the data. It increase the scope of detecting maximum number of proteins with least inter-individual variation within a specific treatment group (Park et al., 2012; Somashekar et al., 2017; Muhammad Aslam et al., 2019). Further experiments involving the data analysis considering higher number of bulls individually (instead of pooling) within a specific group will definitely help in gaining deeper and robust insights into what has been conveyed in the current study.

## 7 Conclusion

In conclusion, our study established the comparative proteomic profiles of high- and low-fertile buffalo bull spermatozoa using label free LC-MS/MS. Many of the proteins which were highly abundant in high fertile bull spermatozoa have roles in spermatogenesis, sperm motility, acrosome integrity, zona pellucida binding and other associated sperm functions. The spermatozoa from low fertile bulls were found to have higher abundance of proteins involved in glycolysis, fatty acid metabolism and inflammation. The differential abundance of AKAP3, DLD, and Sp17 in disntinct fertility groups and their quantification through Western blot and cellular localization through immunocytochemistry strenghthened the findings. Altogether, the differential abundance of these proteins convincingly conveys the reason for contrasting fertilities in high and low fertile bulls. However, the findings in the current study need to be validated on a large number of bulls before using the proteins as fertility markers. Hence, the shortlisted proteins may serve as potential candidates in buffaloes for differentiating the high fertile bull spermatozoa from the low fertile.

## Data Availability

The datasets presented in this study can be found in online repositories. The names of the repository/repositories and accession number(s) can be found below: https://www.ebi.ac.uk/pride/archive/, PXD039680.
