# Supplementary material for: Identification of protein candidates in spermatozoa of water buffalo (Bubalus bubalis) bulls helps in predicting their fertility status
Source: Front Cell Dev Biol. 2023 Feb 20;11:1119220. doi: 10.3389/fcell.2023.1119220 (PMC9986327; doi:10.3389/fcell.2023.1119220)
Supplement: Supplementary file 1 [file Table1.DOCX]

**Supplementary information**

**Table-1 Classification of high and low fertile buffalo bulls.**

| **Bull no.** | **Place** | **AI** | **CR** | **Fertility** |
| --- | --- | --- | --- | --- |
| 4733 | CIRB | 59 | 54 | **HF** |
| 4100 | CIRB | 78 | 56 | **HF** |
| 4807 | NDRI | 104 | 51 | **HF** |
| 5943 | NDRI | 135 | 51 | **HF** |
| 1933 | CIRB | 96 | 51 | **HF** |
| 5720 | NDRI | 62 | 33 | **LF** |
| 3267 | CIRB | 98 | 33 | **LF** |
| 4395 | NDRI | 148 | 32 | **LF** |
| 7010 | NDRI | 56 | 32 | **LF** |
| 4640 | NDRI | 90 | 28 | LF |

**Figure-1 SDS PAGE gel of HF and LF isolated protein.**


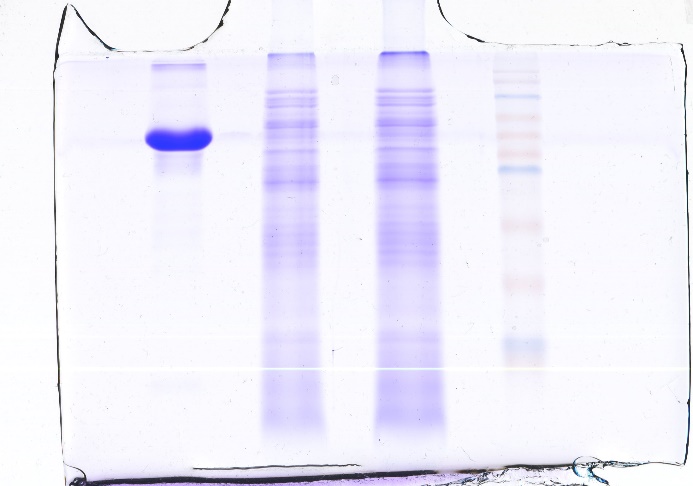


**175 kda**

**130 Kda**

**95 Kda**

**70 Kda**

**62 kda**

**51 kda**

**42 kda**

**29 kda**

**22 kda**

**14 kda**

**10.5 kda**

Ladder

BSA

LF

HF


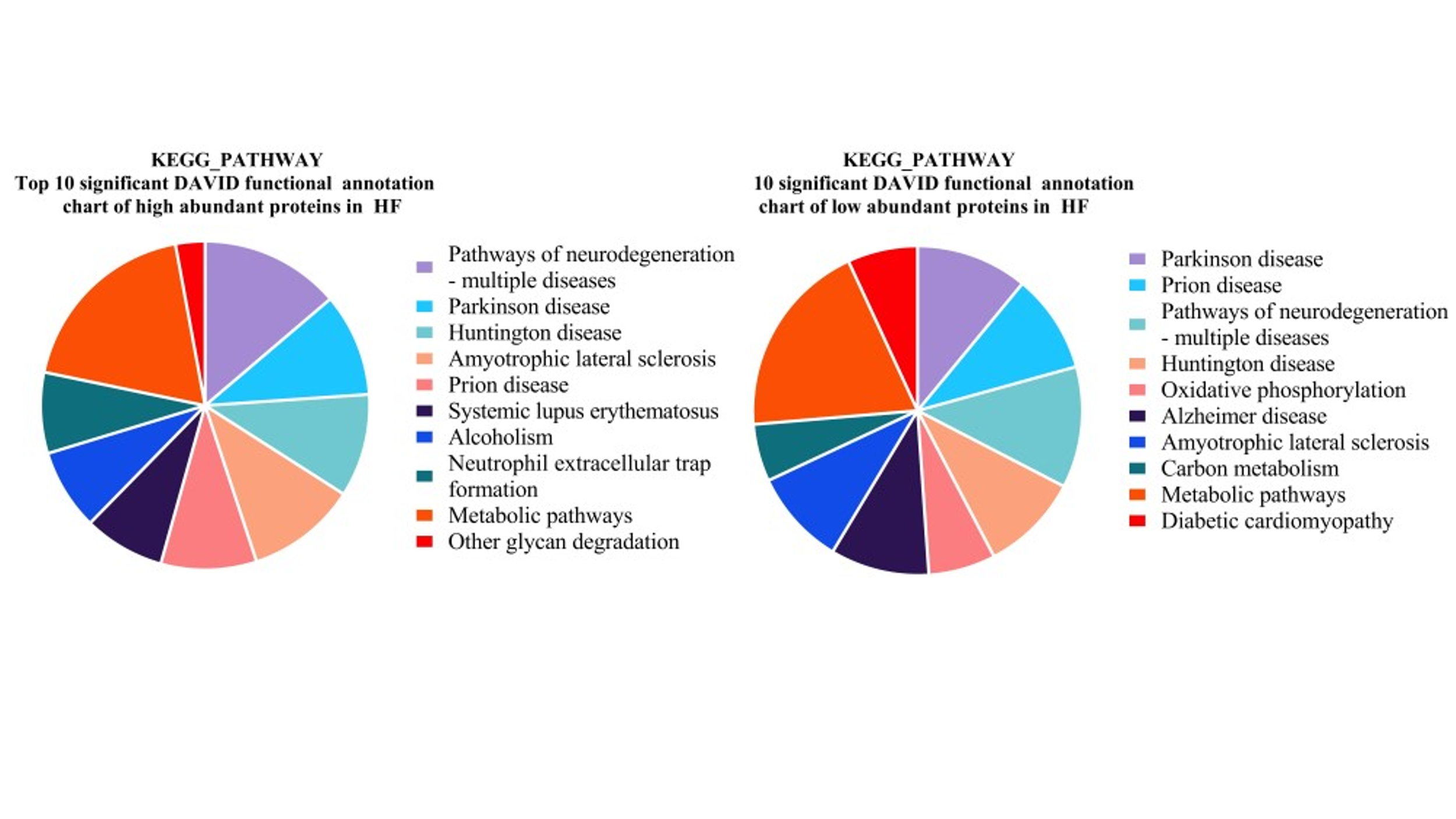
**Figure-2 KEGG Pathways of HF high and low abundant proteins**


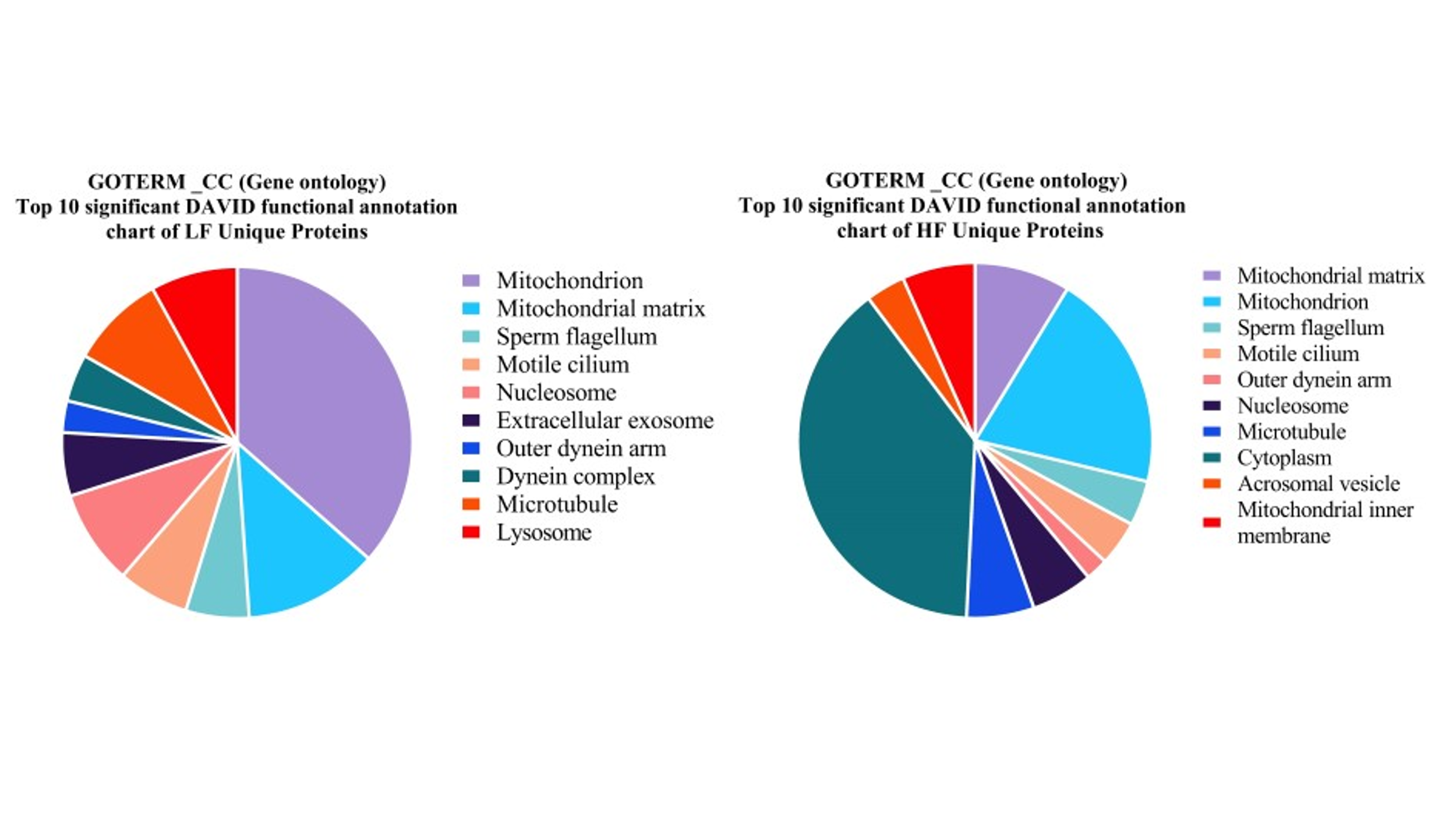

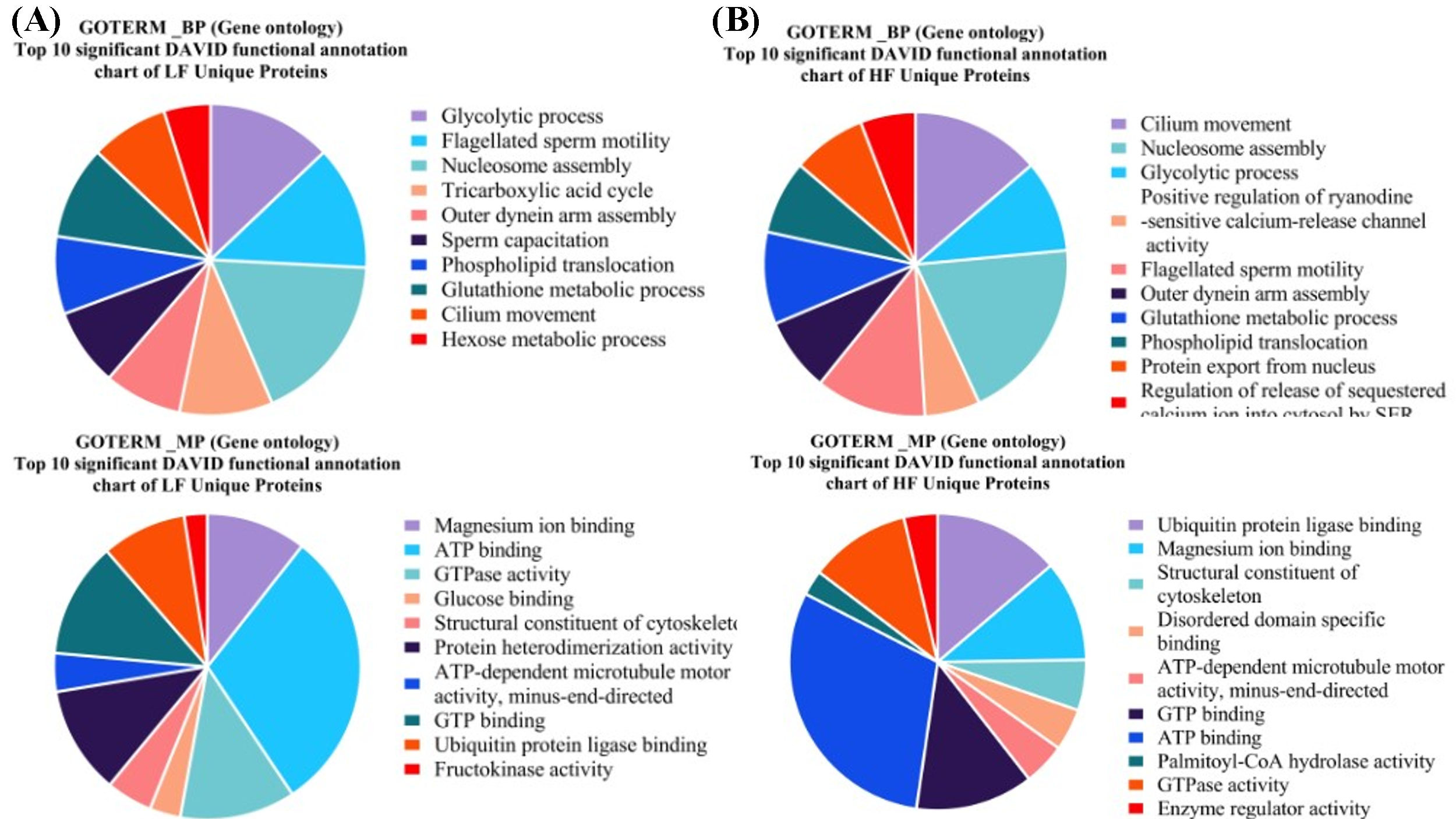
**Figure-3 GO of HF and LF unique proteins**

**Table-2 Gene Ontology and pathway analysis of high and low abundant proteins in HF**

1. **Biological process of high abundant proteins in HF**

| Term | Count | p value | Gene |
| --- | --- | --- | --- |
| GO:0006457~protein folding | 9 | 1.84E-06 | CCT4, PDIA1, LOC613401, P4HB, PDILT, CCT3, PDIA3, PDIA3, CCT8L2, CCT4, P4HB, CCT6B, TRAP1, CCT6B |
| GO:0006342~chromatin silencing | 6 | 1.75E-05 | H2AC20, H2AZ1, H2AZ2, H2AC21 |
| GO:0007283~spermatogenesis | 9 | 2.38E-04 | BCAP31, TDRKH, ODF1, TSGA10, ODF2, SMRP1, SPATA19, OAZ3, SEPTIN12 |
| GO:0030317~flagellated sperm motility | 5 | 0.00124 | TEKT4, PRSS55, TEKT3, TEKT5, AKAP4 |
| GO:1901998~toxin transport | 4 | 0.001627 | CCT4, HSPA5, CCT4, CCT6B, CCT6B, CCT3 |
| GO:0007339~binding of sperm to zona pellucida | 4 | 0.003403 | CCT4, CCT4, PRSS55, CCT3, SPA17 |
| GO:0060294~cilium movement involved in cell motility | 3 | 0.004192 | TEKT4, TEKT3, TEKT5, TEKT5, TEKT3 |
| GO:0007286~spermatid development | 4 | 0.012063 | SPINK2, OSBP2, PDILT, H3-3A, H3-3B |
| GO:0060378~regulation of brood size | 2 | 0.01507 | TEKT4, TEKT3, TEKT3 |
| GO:0051085~chaperone mediated protein folding requiring cofactor | 3 | 0.020167 | HSPA6, HSPA5, DNAJB13 |

1. **Molecular functions of high abundant proteins in HF**

| Term | Count | p value | Genes |
| --- | --- | --- | --- |
| GO:0030527~structural constituent of chromatin | 6 | 7.85E-08 | H3-5, H2AC20, H2AZ1, H3-3A, H3-3B, H2AZ2 |
| GO:0046982~protein heterodimerization activity | 14 | 3.11E-07 | HEXA, P4HB, H2AZ1, H3-3A, H3-3B, H2AZ2, H2AC21, LOC100297725, H3-5, HIP1, P4HB, H2AC20, HIST2H3D |
| GO:0005524~ATP binding | 27 | 1.03E-06 | AK1, TKFC, IQCA1L, HSPA5, PSMC2, NME7, CCT3, PGK1, DARS2, ATP2B2, UBA1, CCT8L2, RUVBL2, HSPA6, CCT4, VRK3, PSMC4, TRAP1, NME7, CCT6B, CCT4, KATNAL2, CSNK2A2, TKFC, PGK1, RUVBL1, GSK3A, CSNK2A2, IQCA1L, RUVBL2, ATP2B1, CCT6B, DNAH12, DARS2, ATP2B3 |
| GO:0016887~ATPase activity | 8 | 1.29E-06 | CCT4, RUVBL2, HSPA5, CCT4, PSMC4, RUVBL2, PSMC2, CCT6B, TRAP1, CCT6B, CCT3 |
| GO:0051082~unfolded protein binding | 8 | 2.03E-06 | HSPA6, CCT8L2, HSPA5, CCT4, DNAJB13, CCT6B, TRAP1, CCT6B, CCT3 |
| GO:0044183~protein binding involved in protein folding | 5 | 4.96E-05 | HSPA6, HSPA5, CCT4, CCT6B, CCT6B, CCT3 |
| GO:1905056~calcium-transporting ATPase activity involved in regulation of presynaptic cytosolic calcium ion concentration | 3 | 1.21E-04 | ATP2B1, ATP2B2, ATP2B3 |
| GO:0003756~protein disulfide isomerase activity | 4 | 1.62E-04 | PDIA6, P4HB, P4HB, PDILT, PDIA3, PDIA3 |
| GO:0017025~TBP-class protein binding | 4 | 3.11E-04 | RUVBL2, CAND2, RUVBL2, PSMC2, RUVBL1 |
| GO:0004563~beta-N-acetylhexosaminidase activity | 3 | 4.00E-04 | HEXA, LOC786974, HEXB |

1. **Cellular component of high abundant proteins in HF**

| Term | Count | p value | Genes |
| --- | --- | --- | --- |
| GO:0000786~nucleosome | 11 | 2.43E-07 | LOC100297725, H3-5A, H2AC20, H2AZ1, H3-3A, H3-3B, HIST2H3D, H2AZ2, H2AC21 |
| GO:0005759~mitochondrial matrix | 10 | 2.16E-06 | PDP1, CLPP, LYRM7, ADPRS, DARS2, LYRM7, ADPRS, PDP1, ALDH2, SIRT5, ALDH4A1, TRAP1, DARS2, FDX2 |
| GO:0005879~axonemal microtubule | 6 | 2.75E-06 | CFAP210, TEKT4, TEKT3, NME7, TEKT3, NME7, MNS1, LOC539526 |
| GO:0036126~sperm flagellum | 6 | 6.45E-06 | TEKT4, ODF2, SPATA19, TEKT3, TEKT5, ODF2, TEKT5, LOC539526 |
| GO:0005832~chaperonin-containing T-complex | 4 | 6.46E-05 | CCT4, CCT8L2, CCT4, CCT6B, CCT6B, CCT3 |
| GO:0031514~motile cilium | 6 | 1.15E-04 | ANXA1, TEKT3, TEKT5, TEKT3, DRC1, MNS1, IQCD |
| GO:0042470~melanosome | 6 | 1.69E-04 | CCT4, TMED10, PDIA6, HSPA5, CCT4, P4HB, PDIA3 |
| GO:0043231~intracellular membrane-bounded organelle | 10 | 3.10E-04 | OSBP2, HSPA5, LOC613401, CYB5A, HIP1, ATP2B1, PHLPP2, CFAP157, ATP2B2, ATP2B3 |
| GO:0005829~cytosol | 36 | 3.96E-04 | AK1, ANXA1, HEXA, TKFC, HSPA5, OSBP2, EXTL2, DNAJB13, CYB5A, P4HB, CDO1, PSMC2, DRC1, PGK1, RUVBL2, HSPA6, PSMC4, PHLPP2, SIRT5, ALDH4A1, CFAP157, CUTC, CSNK2A2, DCTN2, ANXA1, TKFC, RGS22, ALMS1, S100A16, PGK1, RUVBL1, GAPDHS, GSK3A, CSNK2A2, EFR3A, MYL6, CAND2, P4HB, DNPEP, RUVBL2, SCYE1, TPI1 |
| GO:0097228~sperm principal piece | 4 | 4.30E-04 | TEKT4, AKAP3, AKAP3, AKAP4, SPA17 |

1. **Biological process of low abundant proteins in HF**

| Term | Count | P value | Genes |
| --- | --- | --- | --- |
| GO:0010499~proteasomal ubiquitin-independent protein catabolic process | 7 | 7.50E-07 | PSMB7, PSMA4, PSMA6, PSMB4, PSME4, PSMB2, PSMB5 |
| GO:0006099~tricarboxylic acid cycle | 7 | 2.92E-06 | DLAT, SUCLG2, SDHA, IDH2, OGDH, SDHA, SDHB, DLST |
| GO:0006096~glycolytic process | 7 | 3.55E-06 | PFKL, HK3, PGAM2, HK3, ENO3, OGDH, HK1, PGAM2, HK1, GPI |
| GO:0006103~2-oxoglutarate metabolic process | 5 | 6.05E-06 | KYAT3, IDH2, OGDH, DLST, GOT2 |
| GO:1902600~hydrogen ion transmembrane transport | 9 | 6.21E-06 | ATP5F1E, ATP6V1B1, SLC25A5, COX4I1, MGC165862, ATP5MG, ATP6V1E2, ATP5MF, ATP5ME |
| GO:0048240~sperm capacitation | 6 | 1.11E-05 | SLC26A8, PRKACA, SLC26A8, ROPN1, DLD |
| GO:0019563~glycerol catabolic process | 4 | 7.86E-05 | LOC538702, GPD2, GK2, GK |
| GO:0032981~mitochondrial respiratory chain complex I assembly | 7 | 1.44E-04 | ACAD9, BCS1L, NDUFA12, ACAD9, NDUFA11, NDUFA9, NDUFC2, NDUFS8 |
| GO:0015986~ATP synthesis coupled proton transport | 5 | 2.65E-04 | ATP5MG, ATP5ME |
| GO:0019941~modification-dependent protein catabolic process | 4 | 3.56E-04 | RPS27A, UBA52, UBB, UBC |

1. **Molecular functions of low abundant proteins in HF**

| Term | Count | P value | Genes |
| --- | --- | --- | --- |
| GO:0004298~threonine-type endopeptidase activity | 6 | 3.25E-07 | PSMB7, PSMA4, PSMB4, PRSS50, PSMB2, PSMB5, PSMB7 |
| GO:0015078~hydrogen ion transmembrane transporter activity | 7 | 1.13E-06 | ATP6V1B1, SLC25A5, ATP5MG, ATP5ME |
| GO:0004175~endopeptidase activity | 6 | 2.24E-04 | PSMB7, PSMA4, PSMA6, PSMB4, PSMB2, PSMB5, PSMB7 |
| GO:0009055~electron carrier activity | 5 | 3.46E-04 | SDHA, SDHA, ETFA, MT-CO3, COX7A1, SDHA, SDHB |
| GO:0005524~ATP binding | 32 | 3.88E-04 | LOC538702, CSNK1G1, HK3, ACSF3, BCS1L, FLAD1, MYO18A, ENTPD1, HK1, CARS2, ENTPD1, CAMK4, LOC524391, SUCLG2, MMAB, PRKACB, AK3, DNAH11, LOC524391, DGUOK, HK3, UBA6, ATP6V1B1, CSNK1G3, MMAB, GK, CARS2, SRR, CSNK1G1, PCCB, PEBP1, DNAH10, PFKL, PRKACA, GK2, HK1, MYH9E, ATP8B1, PRKACB |
| GO:0050660~flavin adenine dinucleotide binding | 6 | 5.81E-04 | ACAD9, TXNRD1, ACADVL, SDHA, SDHA, ETFA, ACAD9, SDHA, DLD |
| GO:0005525~GTP binding | 14 | 5.96E-04 | RAB14, SEPTIN6, RAB8A, ARL8A RAB2A, TUBB4B, ARL8B, TUBB2A, SUCLG2, SEPTIN11, TUBB2A, RAB11B, SEPTIN8, RHOB, ARL8B, AK3 |
| GO:0004784~superoxide dismutase activity | 3 | 7.52E-04 | SOD2, SOD1, SOD2 |
| GO:0004129~cytochrome-c oxidase activity | 4 | 0.001026 | COX3, MT-CO3, MT-CO2, COX4I1 |
| GO:0004370~glycerol kinase activity | 3 | 0.001244 | LOC538702, GK2, GK |

1. **Cellular component of low abundant proteins in HF**

| Term | Count | P value | Genes |
| --- | --- | --- | --- |
| GO:0005739~mitochondrion | 59 | 9.73E-25 | PDHA2 ,KIFBP, LOC538702, SSBP1, MPST, ACSF3, NDUFA11, NIPSNAP3A, PSMB4, HK1, SSBP1, LACTB, SUCLG2, SLC25A20, MT-CO2, COX7A1, HSD17B10, SDHA, AK3, SOD2, DLD, DGUOK, ACAD9, HK3, SDHA, UBB, PRDX6, OGDH, MMAB, ACAD9, FTMT, PMPCA, DLST, GOT2, ETFA, IDH2, MT-CO3, SOD2, GK2, SLC25A1, FAM162A, ECI1, SDHA, HSDL2, HK3, BRI3BP, ABHD10, NDUFA9, SLC25A3, TXNRD1, MMAB, HSDL2, DYNLL1, NAXE, PARK7, SOD1, UQCRC2, SLC25A1, SLC25A11, ROMO1, GPD2, ME2, GK, CPT1B, ACADVL, KYAT3, PRKACA, HK1, FTMT |
| GO:0005743~mitochondrial inner membrane | 20 | 5.52E-09 | SLC25A11, ATP5F1E, SDHA, COX3, SDHA, SLC25A31, BCS1L, SLC25A5, SLC25A31, PMPCA, SLC25A35, ATP5MF, SDHB, GOT2, SLC25A3, NDUFV1, IDH2, MT-CO3, MT-CO2, SDHA, SLC25A1, SLC25A1, NDUFS8, UQCRC2 |
| GO:0005759~mitochondrial matrix | 14 | 1.86E-07 | DLAT, OGDH, CARS2, CARS2, PMPCA, PCCB, NDUFA9, ISCU, DLST, GOT2, ETFA, FABP3, OD2, PARK7, AK3, SOD2 |
| GO:0005753~mitochondrial proton-transporting ATP synthase complex | 7 | 8.45E-07 | ATP5F1E, ATP5MJ, ATP5MK, ATP5MG, ATP5MF, ATP5ME |
| GO:0005839~proteasome core complex | 6 | 6.44E-06 | PSMB7, PSMA4, PSMA6, PSMB4, PSMB2, PSMB5, PSMB7 |
| GO:0045277~respiratory chain complex IV | 6 | 1.54E-05 | COX3, MT-CO3, MT-CO2, COX7A1, COX4I1, COX7C |
| GO:0031514~motile cilium | 8 | 2.66E-05 | SLC25A31, SEPTIN6, SLC25A31, LDHC, ROPN1, DNAH11, DLD, CFAP20, RSPH1 |
| GO:0000276~mitochondrial proton-transporting ATP synthase complex, coupling factor F(o) | 5 | 3.83E-05 | ATP5MG, ATP5ME |
| GO:0001669~acrosomal vesicle | 7 | 2.04E-04 | C11H9ORF9, LYZL6, PRKACA, LYZL, ACRBP, MROH2B, ACE3, ATP6V1E2 |
| GO:0019774~proteasome core complex, beta-subunit complex | 4 | 2.16E-04 | PSMB7, PSMB4, PSMB2, PSMB5, PSMB7 |

1. **KEGG pathways of high abundant proteins in HF**

| Term | Count | P value | Genes |
| --- | --- | --- | --- |
| bta05022: Pathways of neurodegeneration - multiple diseases | 19 | 5.34E-08 | CSNK2A2, HSPA5, DCTN2, PSMD7, PSMC2, MT-ATP8, NDUFA3, UBA1, CSNK2A2, COX5B, PSMC4, NDUFS6, NDUFB6, HIP1, COX5A, DCTN6, LOC101906363, TRAP1, DNAH12, UCHL1 |
| bta05012: Parkinson disease | 14 | 3.94E-07 | HSPA5, PSMD7, PSMC2, MT-ATP8, NDUFA3, UBA1, COX5B, PSMC4, NDUFS6, NDUFB6, COX5A, LOC101906363, TRAP1, UCHL1 |
| bta05016: Huntington disease | 14 | 1.60E-06 | DCTN2, PSMD7, PSMC2, MT-ATP8, NDUFA3, COX5B, PSMC4, NDUFS6, NDUFB6, HIP1, COX5A, DCTN6, LOC101906363, DNAH12 |
| bta05014: Amyotrophic lateral sclerosis | 15 | 2.19E-06 | HSPA5, DCTN2, HNRNPA1, PSMD7, PSMC2, MT-ATP8, NDUFA3, COX5B, PSMC4, NDUFS6, NDUFB6, COX5A, DCTN6, LOC101906363, DNAH12 |
| bta05020: Prion disease | 13 | 2.70E-06 | CSNK2A2, HSPA5, PSMD7, PSMC2, MT-ATP8, NDUFA3, CSNK2A2, HSPA6, COX5B, PSMC4, NDUFS6, NDUFB6, COX5A, LOC101906363 |
| bta05322: Systemic lupus erythematosus | 11 | 3.04E-06 | LOC100297725, H3-5, H2AC20, H2AZ1, H3-3A, H3-3B, HIST2H3D, H2AZ2, H2AC21 |
| bta05034: Alcoholism | 11 | 1.78E-05 | LOC100297725, H3-5, H2AC20, H2AZ1, H3-3A, H3-3B, HIST2H3D, H2AZ2, H2AC21 |
| bta04613: Neutrophil extracellular trap formation | 11 | 2.39E-05 | LOC100297725, H3-5, H2AC20, H2AZ1, H3-3A, H3-3B, HIST2H3D, H2AZ2, H2AC21 |
| bta01100: Metabolic pathways | 26 | 6.83E-04 | AK1, HEXA, TKFC, EXTL2, CDO1, NME7, MT-ATP8, PGK1, COX5B, COX5A, ACP1, SIRT5, ALDH4A1, LOC786974, NME7, LOC539818, ABHD16A, TKFC, HMOX2, PGK1, GAPDHS, NDUFA3, ACP1, NDUFS6, NDUFB6, ALDH2, ABHD16, ALDH1A2, TPI1, LOC101906363, HEXB |
| bta00511: Other glycan degradation | 4 | 8.36E-04 | LOC107132586, HEXA, LOC786974, HEXB |

1. **KEGG pathways of low abundant proteins in HF**

| Term | Count | p value | Genes |
| --- | --- | --- | --- |
| bta05012:  Parkinson disease | 35 | 4.94E-21 | NDUFA9, TUBB4B, SDHB, TUBB2A, NDUFV1, RPS27A, UBA52, TUBB2A, PSMD13, MT-CO2, COX7A1, PARK7, SDHA, PRKACB, COX4I1, SOD1, PSMB2, PSMB5, UQCRC2, NDUFS8, ATP5F1E, COX3, SDHA, SLC25A31, UBB, NDUFA12, SLC25A5, PSMB7, PRKACA, PSMA6, PRKACB, NDUFC2, COX7C |
| bta05020:  Prion disease | 31 | 4.82E-17 | UQCR10, SDHA, PSMB7, SLC25A31, PSMA4, NDUFA11, PSMB4, NDUFA9, TUBB4B, SDHB, TUBB2A, NDUFV1, TUBB2A, PSMD13, MT-CO2, COX7A1, SDHA, PRKACB, COX4I1, SOD1, PSMB2, PSMB5, UQCRC2, CSNK2B, NUFS8, ATP5F1E, COX3, SDHA, SLC25A31, NDUFA12, SLC25A5, PSMB7, PRKACA, PSMA6, PRKACB, NDUFC2, COX7C |
| bta05022:  Pathways of neurodegeneration - multiple diseases | 38 | 6.44E-16 | UQCR10, SDHA, PSMB7, SLC25A31, PSMA4, NDUFA11, PSMB4, UBC, NDUFA9, TUBB4B, SDHB, TUBB2A, NDUFV1, RPS27A, UBA52, TUBB2A, PSMD13, MT-CO2, HSD17B10, COX7A1, PARK7, SDHA, COX4I1, SOD1, PSMB2, PSMB5, UQCRC2, CSNK2B, NDUFS8, ATP5F1E, COX3, SDHA, SLC25A31, UBB, NDUFA12, SLC25A5, RAB8A, ACTR1A, PSMB7, DNAH10, PSMA6, NDUFC2, COX7C |
| bta05016:  Huntington disease | 31 | 1.67E-15 | UQCR10, SDHA, PSMB7, SLC25A31, PSMA4, NDUFA11, PSMB4, NDUFA9, TUBB4B, SDHB, TUBB2A, NDUFV1, TUBB2A, PSMD13, MT-CO2, COX7A1, SDHA, COX4I1, SOD1, SOD2, PSMB2, PSMB5, UQCRC2, NDUFS8, ATP5F1E, COX3, SDHA, SLC25A31, NDUFA12, SLC25A5, ACTR1A, PSMB7, DNAH10, SOD2, PSMA6, NDUFC2, COX7C |
| bta00190:  Oxidative phosphorylation | 21 | 7.63E-14 | UQCR10, ATP5F1E, SDHA, COX3, SDHA, ATP6V1B1, NDUFA12, NDUFA11, ATP5MG, NDUFA9, ATP6V1E2, ATP5MF, SDHB, ATP5ME, NDUFV1, MT-CO2, COX7A1, SDHA, COX4I1, NDUFC2, COX7C, NDUFS8, UQCRC2 |
| bta05010:  Alzheimer disease | 31 | 7.96E-13 | UQCR10, SDHA, PSMB7, SLC25A31, PSMA4, NDUFA11, PSMB4, NDUFA9, TUBB4B, SDHB, TUBB2A, NDUFV1, TUBB2A, PSMD13, MT-CO2, HSD17B10, COX7A1, SDHA, COX4I1, PSMB2, PSMB5, UQCRC2, CSNK2B, NDUFS8, ATP5F1E, COX3, SDHA, SLC25A31, NDUFA12, SLC25A5, PSMB7, RTN3, PSMA6, NCSTN, NDUFC2, COX7C |
| bta05014:  Amyotrophic lateral sclerosis | 30 | 1.42E-12 | UQCR10, SDHA, PSMB7, PSMA4, NDUFA11, PSMB4, NDUFA9, TUBB4B, SDHB, TUBB2A, NDUFV1, TUBB2A, PSMD13, MT-CO2, COX7A1, SDHA, COX4I1, SOD1, PSMB2, PSMB5, UQCRC2, NDUFS8, ATP5F1E, COX3, SDHA, NUP205, NDUFA12, RAB8A, ACTR1A, PSMB7, DNAH10, PSMA6, NDUFC2, COX7C |
| bta01200:  Carbon metabolism | 18 | 1.63E-12 | DLD, PDHA2, DLAT, SDHA, SDHA, ENO3, OGDH, ME2, PGAM2, HK1, GPI, PCCB, DLST, SDHB, GOT2, FBP1, PFKL, SUCLG2, PGAM2, IDH2, HK1, SDHA |
| bta01100:  Metabolic pathways | 62 | 4.05E-12 | PDHA2, LOC538702, MPST, ACSF3, NDUFA11, ENTPD1, HK1,GLB1, ENTPD1, ATP5MG, ATP6V1E2, ATP5MF, SDHB, ATP5ME, SUCLG2, MT-CO2, COX7A1, HSD17B10, SDHA, COX4I1, ISYNA1, DLD, DGUOK, NDUFS8, DLAT, SDHA, AKR1A1, ATP6V1B1, PRDX6, OGDH, MMAB, PGAM2, GPI, PCCB, DLST, GOT2, PFKL, PGAM2, IDH2, LDHC,GK2,GAA, UQCR10, SDHA, ENO3, FLAD1, GLB1, NDUFA9, NDUFV1, MMAB, CA2, UQCRC2, ATP5F1E, COX3, STT3B, NDUFA12, FDPS, GK, SRR, AHCY, PLCZ1, FBP1, ACADVL, CA2, KYAT3, ACOT2, HK1, NDUFC2, COX7C |
| bta05415:  Diabetic cardiomyopathy | 22 | 2.16E-11 | UQCR10, PDHA2, SDHA, PPP1CB, SLC25A31, NDUFA11, NDUFA9, SDHB, NDUFV1, MT-CO2, COX7A1, SDHA, COX4I1, NDUFS8, UQCRC2, ATP5F1E, COX3, SDHA, SLC25A31, NDUFA12, SLC25A5, CPT1B, ACE3, NDUFC2, COX7C |
