# Supplementary figures and images for: Identification of protein candidates in spermatozoa of water buffalo (Bubalus bubalis) bulls helps in predicting their fertility status
Source: Front Cell Dev Biol. 2023 Feb 20;11:1119220. doi: 10.3389/fcell.2023.1119220 (PMC9986327; doi:10.3389/fcell.2023.1119220)

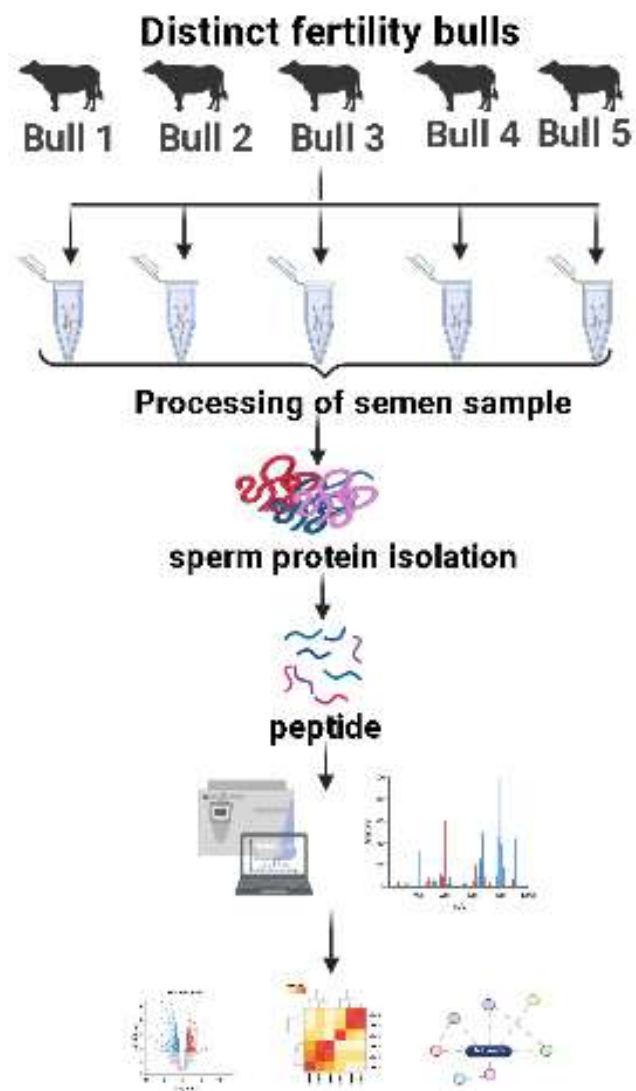

Supplement: Supplementary file 2 [file Presentation1.PDF]
